# Senescence-circadian interplay stratifies patient prognosis and reveals immune remodeling heterogeneity in colorectal cancer

**DOI:** 10.3389/fimmu.2026.1804974

**Published:** 2026-06-03

**Authors:** Li-zhi Luo, Yu Zhan, Ru-xiang Bao, Yong-qiang Zheng, Qi-nian Wu, Shao-yan Xi, Ze-xian Liu, Ting Li, Qi Zhao

**Affiliations:** 1 State Key Laboratory of Oncology in South China, Guangdong Provincial Clinical Research Center for Cancer, Sun Yat-sen University Cancer Center, Guangzhou, China; 2 Department of Bioinformatics, Sun Yat-sen University Cancer Center, Guangzhou, China; 3 Department of Gastroenterology, The Affiliated Cancer Hospital of Xiangya School of Medicine, Central South University/Hunan Cancer Hospital, Changsha, China

**Keywords:** cellular senescence, circadian rhythm, colorectal cancer, immune heterogeneity, NOX4, prognostic risk score, single-cell RNA sequencing

## Abstract

**Background:**

Colorectal cancer (CRC) exhibits substantial biological and prognostic heterogeneity that is not fully captured by conventional clinicopathological staging, and the interplay between the cellular senescence and circadian dysregulation in CRC remains insufficiently defined.

**Methods:**

We integrated bulk transcriptomic and clinical data from public CRC cohorts to construct a senescence-circadian interplay score (SCore). Senescence-related and circadian-related gene sets were intersected with CRC differentially expressed genes to identify candidate genes, from which a four-gene random survival forest model was established. The model was evaluated in the TCGA-COAD/READ training cohort and externally validated in GSE12945 and GSE39582. We further characterized clinicopathological associations, prognostic independence, nomogram performance, pathway enrichment, consensus molecular subtype distribution, immune landscape features, and predicted drug sensitivity. Single-cell RNA sequencing dataset was used to localize SCore-associated programs and to examine T-cell communication and differentiation dynamics. In addition, RT-qPCR in CRC cell lines, immunohistochemical validation in 120 paired CRC and adjacent non-tumor tissues, and NOX4-centered knockdown experiments were performed to provide orthogonal experimental support.

**Results:**

A higher SCore was consistently associated with poorer overall survival across cohorts and retained prognostic value when integrated with clinicopathological variables. High-SCore tumors were characterized by enrichment of oxidative stress, extracellular matrix remodeling, focal adhesion, and invasion-related programs, together with computationally inferred immune dysfunction/exclusion-associated features. Single-cell analyses localized SCore-associated signals to a T-cell-centered context, where cell–cell communication, pseudotime dynamics, and functional-state remodeling converged. Notably, MIF-(CD74+CXCR4) signaling emerged as a prominent interaction axis. Consistent with the transcriptomic findings, immunohistochemistry confirmed higher expression of NOX4, CXCL1, CDKN2A, and SIX1 in CRC tissues than in paired adjacent non-tumor tissues. Functionally, NOX4 knockdown reduced intracellular ROS, attenuated multiple inflammatory/immunoregulatory mediators, lowered PD-L1 protein expression, and suppressed migratory capacity, supporting a NOX4-associated redox/inflammatory component within the broader SCore-linked state.

**Conclusion:**

SCore provides biologically interpretable transcriptomic framework for CRC risk stratification. The NOX4-centered data provide functional support for one component of this state, whereas the broader immune and circadian implications remain hypothesis-generating.

## 1 Introduction

Colorectal cancer (CRC) is among the most prevalent malignancies of the digestive system and remains a leading cause of cancer-related morbidity and mortality worldwide (1–4). Despite substantial advances in screening, surgery, and systemic treatment, CRC continues to exhibit marked biological and clinical heterogeneity, resulting in considerable variability in recurrence risk, metastatic behavior, treatment response, and long-term outcome. The increasing incidence of early-onset CRC further highlights limitations in current screening strategies and risk-assessment paradigms (5, 6). Although contemporary clinical management is guided by tumor stage and selected molecular features, these parameters do not fully explain interpatient heterogeneity. In particular, while immune checkpoint blockade provides durable benefit for a subset of microsatellite instability-high tumors, most microsatellite-stable CRCs derive limited benefit from immunotherapy (7–10). Improved molecular stratification strategies are therefore needed to better capture clinically relevant heterogeneity beyond conventional staging and to support more individualized prognostic assessment and biological stratification (11).

Cellular senescence is a stress-responsive state characterized by durable cell-cycle arrest and acquisition of a senescence-associated secretory phenotype (SASP). Although senescence may initially function as an intrinsic barrier to malignant transformation, persistent senescent-cell accumulation and prolonged SASP signaling can also reshape the tumor microenvironment (TME), sustain chronic inflammation, and promote malignant progression in established tumors (12, 13). In CRC, senescence-associated tumor-cell states have been linked to invasive behavior and metastatic potential (14). In addition, SASP-related programs contribute to tumor-immune remodeling by altering inflammatory signaling and immune-cell recruitment within the TME (15–17).

Circadian rhythm is an endogenous timing system that coordinates diverse biological processes relevant to tumor biology, including metabolism, cell-cycle control, DNA damage repair, redox homeostasis, and immune regulation (18–20). Increasing evidence indicates that circadian disruption contributes to CRC pathogenesis and progression, and aberrant expression of clock-related genes has been associated with aggressive clinicopathological features and unfavorable outcome (21–24). Importantly, circadian regulation and cellular senescence are not independent processes, but are mechanistically interconnected through shared stress-response, redox, and inflammatory circuitry. Core clock components such as CLOCK and BMAL1 have been implicated in DNA damage responses, oxidative-stress regulation, and SASP-associated inflammatory signaling, thereby influencing the establishment and maintenance of senescent states (25–29). Conversely, both oxidative stress-induced premature senescence and replicative senescence have been associated with altered circadian properties, including phase delay, period lengthening, reduced circadian amplitude, and tissue-level circadian reprogramming (28, 30–33). Together, these observations support a bidirectional regulatory relationship between senescence and circadian control, raising the possibility that their transcriptional overlap may mark a biologically meaningful stress-adaptive state relevant to tumor progression and immune remodeling in CRC (17, 20, 22, 29).

However, most previous studies have examined senescence-associated programs and circadian dysregulation separately. As a result, whether their overlapping transcriptional space captures a clinically and biologically relevant state in CRC remains insufficiently defined. Addressing this question requires both population-level robustness and cellular-level resolution. Bulk transcriptomic cohorts linked to survival outcomes are well suited for identifying stable prognostic patterns, whereas single-cell RNA sequencing (scRNA-seq) can localize risk-associated programs to specific cellular compartments and dynamic states within the tumor microenvironment (34–38).

In this study, we integrated bulk and single-cell transcriptomic data from public CRC cohorts to investigate the prognostic and biological relevance of genes residing within the senescence- and circadian-related transcriptional overlap. We constructed and externally validated a four-gene random survival forest-derived senescence-circadian interplay score (SCore), and then characterized its associated pathways, clinicopathological correlations, immune-related features, and cellular context using bulk RNA-seq and scRNA-seq analyses. To provide orthogonal support for the model-associated genes, we performed RT-qPCR in CRC cell lines and immunohistochemical validation in paired CRC and adjacent non-tumor tissues, followed by siRNA-mediated NOX4 knockdown assays to examine a redox/inflammatory component of the SCore-associated state. Importantly, SCore is interpreted here as a transcriptomic index reflecting senescence-circadian transcriptional overlap, rather than a direct quantitative readout of mechanistic senescence-circadian coupling. Together, these analyses establish a reproducible risk-scoring framework associated with stress-adaptive and immune-related heterogeneity in CRC.

## 2 Materials and methods

### 2.1 Data acquisition

The training cohort used in this study was obtained from The Cancer Genome Atlas (TCGA) database (https://portal.gdc.cancer.gov). Transcriptomic profiles (RNA sequencing), somatic mutation data, and corresponding clinical information were downloaded for patients with colorectal cancer. The TCGA-COAD and TCGA-READ datasets were merged to generate a unified CRC cohort. In total, 618 CRC tumor tissue samples and 51 adjacent non-tumor tissue samples were included, among which 524 tumor samples had complete survival information and were retained for subsequent prognostic analyses. An independent validation cohort was retrieved from the Gene Expression Omnibus (GEO) database (https://www.ncbi.nlm.nih.gov/geo/). The GSE12945 dataset (platform: GPL96) was selected as the external validation set and consisted of 62 CRC tumor samples with available survival data. In addition, another independent external validation dataset, GSE39582 (platform: GPL570), was downloaded from the GEO database. It contained 556 colorectal cancer tumor tissue samples (microarray data) with complete survival information. This dataset was used to further validate the robustness of the risk model. For single-cell transcriptomic analysis, the scRNA-seq dataset GSE132465 (platform: GPL20301) was obtained from the GEO database. This dataset comprised 23 CRC tumor tissue samples and 10 adjacent non-tumor tissue samples and was used to characterize the cellular distribution and expression dynamics of prognostic genes at single-cell resolution. Gene sets related to cellular senescence and circadian rhythm were compiled from previously published studies. The senescence-related gene set was obtained from the CellAge database (https://genomics.senescence.info/cells/) and consisted of 278 cellular senescence-associated genes (39). The circadian rhythm-related gene set was retrieved from the GeneCards database (https://www.genecards.org/) by searching with the keyword “circadian rhythm”, and genes with a relevance score below 0.4 were excluded, resulting in a final set of 2,095 genes (40). Intersecting these two curated gene sets yielded 75 overlapping genes, hereafter referred to as senescence-circadian associated (ACR) genes (**Supplementary Table 1**). These genes were retained as the initial candidate set for subsequent analyses. All datasets were accessed on August 7, 2025. The detailed study workflow was shown in **Supplementary Figure 1**.

### 2.2 Analysis of differential gene expression

Differentially expressed genes (DEGs) between CRC tumor tissues and adjacent non-tumor tissues in the training cohort were identified using the DESeq2 package (version 1.40.2) in R (41). Genes meeting the criteria of |log_2_ fold change (FC)| > 2 and adjusted P value < 0.05 were considered statistically significant DEGs. The ggplot2 package (version 3.5.2) was used to generate a volcano plot to visualize the overall distribution of DEGs, with genes ranked according to their log_2_FC values (42). The top 10 upregulated and top 10 downregulated genes were annotated on the volcano plot based on fold-change magnitude. In addition, the ComplexHeatmap package (version 2.15.0) was applied to construct a heatmap illustrating the expression patterns of the same top 10 upregulated and top 10 downregulated genes across tumor and normal samples (43).

### 2.3 Identification and related functional analysis of candidate genes

The ggvenn package (version 0.1.10) was used to assess the overlap among differentially expressed genes (DEGs), cellular senescence-related genes, and circadian rhythm-related genes (44). Intersecting these 75 ACR genes with the 2,056 CRC DEGs yielded 10 candidate genes—AGT, BLK, CDKN2A, CXCL1, GATA4, IGFBP1, IL1A, MYLK, NOX4, and SIX1—which were retained for downstream analyses. To explore the biological functions and signaling pathways associated with the candidate genes, Gene Ontology (GO) and Kyoto Encyclopedia of Genes and Genomes (KEGG) enrichment analyses were performed using the clusterProfiler package (version 4.10.1) in R (45). GO enrichment analysis was conducted across three categories, including biological process (BP), cellular component (CC), and molecular function (MF). Terms with a P value < 0.05 were considered statistically significant. To further investigate potential interactions among candidate genes at the protein level, protein-protein interaction (PPI) information was retrieved from the Search Tool for the Retrieval of Interacting Genes (STRING) database (https://string-db.org/). An interaction score threshold greater than 0.15 was applied to construct the PPI network. The resulting network was visualized using the circlize package (version 0.4.10) in R (46).

### 2.4 Discernment of prognostic genes and development of a risk model

Univariate Cox proportional hazards regression analysis was performed on the candidate genes using the survival package (version 3.8.3) (47). This analysis was conducted in 524 CRC tumor samples with complete survival information from the training cohort. Genes with P < 0.05 in univariate Cox regression were considered significantly associated with overall survival. Subsequently, the proportional hazards (PH) assumption was evaluated for the survival-associated genes using the cox.zph function implemented in the survival package (version 3.8.3). Genes that passed the PH assumption test were classified as prognostic genes. Building on this foundation, the random survival forest (RSF) model was introduced to delineate the nonlinear impact of multiple genes on survival outcomes. The RSF model constructed multiple survival trees through random sampling with replacement from the original samples. During the node-splitting process, subsets of predictor variables were randomly selected, with the cumulative hazard function (CHF) serving as the evaluation metric. The ensemble CHF, formed by averaging the CHFs of individual survival trees, was then utilized for individual risk assessment. For patient x, the risk function at time t was derived by averaging the predictions from all survival trees. The formula for h(t|x) was presented as follows: $\hat{H}(\text{t}|\text{x})=\frac{1}{B}\sum_{i=1}^{B}{\hat{H}}_{i}(t|x)$. The CHF at time t given the covariate x was represented as h(t|x), and B denoted the total number of trees within the RSF model. The RSF model was constructed using the randomForestSRC package (v 3.4.1) based on the identified prognostic genes (48). The model parameters were set as ntree = 1,000 and mtry = 4. The senescence-circadian interplay score (SCore) was calculated for each patient in both the training cohort and the external validation cohort (GSE12945) using the established RSF model. An optimal cutoff value for the SCore was determined, which enabled stratification of patients in the training cohort into a high-SCore group (HSG) and a low-SCore group (LSG). The ggplot2 package (version 3.5.2) was used to visualize the distribution of SCore and survival status between the two groups. Kaplan-Meier survival analysis was performed to compare overall survival between the HSG and LSG using the survminer package (version 0.5.0) (49, 50). Time-dependent receiver operating characteristic (ROC) analysis was conducted to evaluate the predictive performance of the prognostic model. ROC curves at 1-year, 2-year, and 3-year time points were generated using the survivalROC package (version 1.0.3.1) (51), and an area under the curve (AUC) value greater than 0.6 was considered indicative of acceptable predictive accuracy. The stability, reliability, and generalizability of the model were further assessed in an independent validation cohort (GSE12945, n = 62, GSE39582, n = 556). SCore was calculated using the same formula and preprocessing pipeline as in the training cohort, whereas the cutoff for defining high- and low-SCore groups was determined within each cohort using the same cutoff-selection procedure.

### 2.5 Co-expression and pathway regulation analysis of core genes

To validate the transcriptional regulatory relationships between the four core genes and oxidative stress- and inflammation-related pathways, a systematic co-expression and pathway activity analysis was performed in TCGA colorectal cancer tumor samples. First, Spearman rank correlation analysis was used to calculate the pairwise expression correlations among the four core genes. A correlation coefficient matrix and raw P values were obtained, and the Benjamini-Hochberg (BH) method was applied to adjust the P values for multiple testing, with an adjusted P < 0.05 considered statistically significant. The results were presented as a correlation heatmap. Second, the official gene sets for the oxidative stress pathway (HALLMARK_REACTIVE_OXYGEN_SPECIES_PATHWAY) and the inflammatory response pathway (HALLMARK_INFLAMMATORY_RESPONSE) were obtained from the Hallmark gene set collection in the MSigDB database. For each core gene, Spearman rank correlation analysis was performed to assess its expression correlation with all qualified genes within each pathway, yielding correlation coefficients (rho) and raw P values for each gene pair. The BH method was then applied to adjust all P values in batch to obtain FDR values. The threshold for significant co-expression was defined as |rho| ≥ 0.3 and FDR < 0.05. The number of significantly co-expressed genes meeting this criterion for each core gene in each pathway was counted and visualized as a grouped bar plot. Finally, the Gene Set Variation Analysis (GSVA) algorithm was used to calculate the enrichment scores of the two pathways in each tumor sample. The expression levels of the four core genes were separately correlated with the corresponding pathway GSVA scores using Spearman rank correlation analysis, and the correlation coefficients and P values were calculated and adjusted by the BH method. The degree of association between core gene expression and pathway activity was displayed as a correlation heatmap, with the same significance criteria as described above.

### 2.6 Association analysis between SCore and clinicopathological characteristics

Based on CRC tumor samples with complete survival information in the training cohort, subgroup analyses were conducted according to clinicopathological characteristics to explore the association between the SCore and clinical variables. Age was dichotomized at 60 years for subgroup analyses. Sex was categorized as male or female. Tumor staging-related variables included T stage, M stage, and overall pathological stage. Differences in SCore distributions among subgroups defined by these clinicopathological characteristics were evaluated using the Wilcoxon rank-sum test or Wilcoxon signed-rank test, as appropriate. A P value < 0.05 was considered statistically significant. To systematically evaluate the association between the SCore and clinicopathological characteristics, we constructed an integrated matrix that included risk groups (high-risk vs. low-risk) and multiple clinical variables. The variables incorporated were sex, age (<60 years vs. ≥60 years), tumor location (left colon, right colon, transverse colon, rectum), MSI status (MSI-H, MSI-L, MSS), histological type (colon adenocarcinoma, colon mucinous adenocarcinoma, rectal adenocarcinoma, rectal mucinous adenocarcinoma), T stage, M stage, and overall pathological stage. Categorical variables were compared between groups using the Chi-square test (or Fisher’s exact test when the expected frequency was <5), while continuous variables (e.g., risk score) were compared using the independent samples t-test. All statistical analyses were performed using the table one package (version 0.13.2) in R software (version 4.3.3), and P < 0.05 was considered statistically significant.

### 2.7 Independent prognostic analysis and construction of nomogram

To further assess the independent prognostic value of the SCore and to identify additional prognostic factors in CRC, Cox proportional hazards regression analyses were performed using the survival package (version 3.8.3). Tumor samples with complete survival information from the training cohort were included in the analysis. Variables incorporated into the univariate Cox regression analysis included SCore, age, sex, overall stage, T stage, and M stage. Factors with P < 0.05 in univariate Cox regression were considered statistically significant. Variables that met the criteria in the univariate Cox analysis were subsequently subjected to proportional hazards (PH) assumption testing (P > 0.05). Eligible variables were then entered into a multivariate Cox regression analysis, followed by an additional PH assumption test (P > 0.05). Factors that remained statistically significant in the multivariate analysis and satisfied the PH assumption were defined as independent prognostic factors. Based on the identified independent prognostic factors, a prognostic nomogram was constructed using the regplot package (version 1.1) (52). Calibration curves were generated using the rms package (version 6.8.1) to assess the agreement between nomogram-predicted survival probabilities and observed outcomes (53). In addition, time-dependent receiver operating characteristic (ROC) curves at 1-, 2-, and 3-year time points were plotted using the survivalROC package (version 1.0.3.1) to evaluate the discriminative performance of the nomogram.

### 2.8 Somatic mutation analysis and identification of consensus molecular subtypes

To investigate the relationship between somatic mutation patterns and risk stratification, tumor mutational burden (TMB) was calculated for CRC samples in the high-SCore group (HSG) and low-SCore group (LSG) using the maftools package (version 2.18.0) (54). Waterfall plots were generated to visualize the top 20 genes with the highest mutation frequencies in the HSG and LSG, respectively. In addition, CRC tumor samples with complete survival information from the training cohort were classified into consensus molecular subtypes (CMS) using the CMSclassifier package (version 2.0.1) (55). According to the CMS classification system, samples were assigned to one of four subtypes: CMS1 (microsatellite instability immune subtype), CMS2 (canonical subtype), CMS3 (metabolic subtype), or CMS4 (mesenchymal subtype). Differences in SCore distributions among the CMS subtypes were subsequently compared using the Wilcoxon test, with a P value < 0.05 considered statistically significant.

### 2.9 Gene set enrichment analysis

Gene set enrichment analysis (GSEA) was performed on CRC tumor samples with complete survival information from the training cohort to systematically investigate signaling pathways associated with different risk states and prognostic genes. Within the training set, differential gene expression between the HSG and LSG was assessed using the DESeq2 package (version 1.40.2), and log_2_ FC values were calculated for all genes. Genes were subsequently ranked in descending order according to their log_2_FC values. The curated gene set c2.cp.kegg_legacy.v2025.1.Hs.symbols was retrieved from the Molecular Signatures Database (MSigDB; https://www.gsea-msigdb.org/) and used as the reference background. GSEA was conducted using the clusterProfiler package (version 4.10.1), with the criteria of |normalized enrichment score (NES)| > 1 and P < 0.05 considered statistically significant. In addition, Spearman correlation coefficients between prognostic genes and all other genes were calculated using the psych package (version 2.2.9). Genes were ranked in descending order based on their correlation coefficients. Under the same KEGG gene set background (c2.cp.kegg_legacy.v2025.1.Hs.symbols), GSEA was further performed for each prognostic gene individually using the clusterProfiler package (version 4.10.1), applying identical significance thresholds (|NES| > 1 and P < 0.05).

### 2.10 Immune microenvironment analysis

CRC tumor samples with complete survival information from the training cohort were used to investigate the association between risk stratification and features of the tumor immune microenvironment. The single-sample gene set enrichment analysis (ssGSEA) algorithm was implemented using the GSVA package (version 1.46.0) (56) to calculate enrichment scores for 28 immune cell types based on previously reported immune-related gene signatures (57). Differences in immune cell infiltration levels between the HSG and LSG were evaluated using the Wilcoxon test, with P < 0.05 considered statistically significant. Spearman correlation analysis was conducted using the psych package (version 2.2.9) to assess associations among differentially infiltrated immune cell types and between immune cell infiltration levels and prognostic gene expression. Correlations with an absolute correlation coefficient greater than 0.30 and a P value < 0.05 were considered statistically significant. Furthermore, the expression levels of 38 previously reported immune checkpoint-related molecules were compared between the HSG and LSG using the Wilcoxon test (P < 0.05) (58). To further evaluate the potential association between risk stratification and immunotherapy response, gene expression profiles of the HSG and LSG were converted to transcripts per million (TPM) format and uploaded to the Tumor Immune Dysfunction and Exclusion (TIDE) database (http://tide.dfci.harvard.edu/). With “Human” selected as the species, TIDE scores were obtained, and differences in TIDE scores between the HSG and LSG were compared using the Wilcoxon test (P < 0.05).

### 2.11 Drug sensitivity analysis

CRC tumor samples with complete survival information from the training cohort were included in the drug sensitivity analysis. Information on CRC-related chemotherapeutic agents was obtained from the Genomics of Drug Sensitivity in Cancer (GDSC) database (https://www.cancerrxgene.org/). The pRRophetic package (version 0.5) was used to predict the half maximal inhibitory concentration (IC_50_) values of individual chemotherapeutic drugs for each tumor sample based on gene expression profiles (59). Differences in predicted IC_50_ values between the HSG and LSG were assessed using the Wilcoxon test, with P < 0.05 considered statistically significant.

### 2.12 Processing of scRNA-seq data and identification of key cell types

Single-cell RNA sequencing data from the GSE132465 dataset were processed and analyzed using the Seurat package (version 5.3.0) (59). Quality control was performed using the PercentageFeatureSet function. Cells meeting the following criteria were retained: 200 < nFeature_RNA < 2,000, nCount_RNA < 10,000, and percent.mt < 10%. In addition, genes detected in fewer than three cells were excluded from downstream analyses. The filtered data were normalized using the NormalizeData function, in which gene expression values for each cell were scaled by total expression, multiplied by a factor of 10,000, and log-transformed. Highly variable genes (HVGs) were identified using the FindVariableFeatures function, and the top 2,000 genes with the highest variability were selected. Data scaling was performed using the ScaleData function, followed by principal component analysis (PCA) using the RunPCA function.

Principal components (PCs) were selected for downstream analyses based on the combined results of the Jackstraw permutation test and Elbow Plot analysis, with PCs showing P < 0.05 and lying within the plateau phase of variance explained retained. Unsupervised clustering was subsequently conducted using the FindNeighbors and FindClusters functions, with a resolution parameter set to 0.1. Dimensionality reduction and visualization were performed using t-distributed stochastic neighbor embedding (t-SNE) via the RunTSNE function. Cell clusters were annotated manually according to established cell-type marker genes reported in the literature (60), enabling identification of CRC-associated cell types. The proportional distribution of each cell type between CRC tumor tissues and adjacent non-tumor tissues was further analyzed. Expression differences of prognostic genes between tumor and normal samples were compared across cell types using the Wilcoxon test. Among the annotated cell populations, T cells were selected as the key cell type for downstream analyses because they represented a major immune compartment in CRC tissues and met the predefined criteria for key-cell selection, including significant tumor-versus-normal differential expression of at least two prognostic genes. Final prioritization was further supported by the convergence of risk-associated gene-expression shifts, intercellular communication patterns, and pseudotime dynamics in this compartment.

To further dissect T-cell heterogeneity within the colorectal cancer tumor microenvironment, all annotated T cells were extracted from the single-cell RNA sequencing data for independent re-clustering analysis. These cells were subjected to independent data normalization, dimensionality reduction by principal component analysis, and unsupervised reclustering analysis using the FindClusters function in the Seurat package with a resolution parameter set to 0.5. Functional annotation of the identified distinct key cell subpopulations was performed using well−recognized canonical markers in the field of tumor immunology. The markers used for annotation included CD4 and IL7R (for CD4+ conventional T cells), CD8A and GZMB (for CD8+ cytotoxic T cells), FOXP3 and IL2RA (for regulatory T cells, Tregs), CCR7 and SELL (for naive T cells), MKI67 and TOP2A (for proliferating T cells), and S100A4 and CD44 (for memory T cells). In addition, the AddModuleScore function was used to calculate the combined expression level of the SCore core gene set (CXCL1, CDKN2A, NOX4, SIX1) in each cell, and the expression distribution of these genes across different cell subpopulations was compared.

### 2.13 Cellular communication and pseudo-temporal trajectory analyses

Cell-cell communication analysis was performed on the GSE132465 scRNA-seq dataset using the CellChat package (version 1.6.1) (60). This approach was applied to quantify and compare the number and strength of intercellular interactions among annotated cell types in both CRC tumor and adjacent normal tissue samples. Ligand-receptor interaction pairs between key cell types and other annotated cell populations were further inferred and visualized, and communication probabilities were calculated to characterize signaling patterns across cell types. For downstream analyses, dimensionality reduction and clustering of key cell types were conducted using the same preprocessing and clustering strategies described for the scRNA-seq data. To reconstruct differentiation trajectories of key cell types, pseudotemporal analysis was performed using the monocle package (version 5.30) (61) in combination with the DDRTree algorithm implemented in the DDRTree package (version 0.1.5) (62). Based on the inferred pseudotime trajectories, dynamic expression patterns of prognostic genes were visualized along pseudotime to characterize their temporal expression changes during cellular differentiation.

### 2.14 Key cell subpopulation re-clustering and dynamic analysis of risk score

To deeply dissect the functional heterogeneity of the risk score (SCore) in key cell populations, key cell subpopulations (celltype == “T cells”) were extracted from the annotated single-cell object, and re-clustering analysis was performed using the Seurat package (5.3.0). The analytical workflow included data normalization (NormalizeData), identification of highly variable genes (FindVariableFeatures), data scaling (ScaleData), principal component analysis (RunPCA), graph-based clustering (FindNeighbors/FindClusters), and t-SNE visualization (RunTSNE). Subsequently, based on the previously constructed risk model gene set, the AddModuleScore function was used to calculate the risk score for each key cell, and cells were divided into high-SCore and low-SCore groups according to the median score.

The Wilcoxon test was employed to compare expression differences of exhaustion-related markers (PDCD1, LAG3, HAVCR2, CTLA4) and senescence-related markers (CDKN2A, TERT, IL6R) between the two groups. The distribution of SCore across key cell populations was visualized using t-SNE feature plots. To explore the relationship between SCore and the differentiation dynamics of key cells, the Monocle package (2.30.1) was used to construct pseudotime trajectories. SCore was mapped onto the trajectory, and trajectories were reconstructed separately for the high- and low-SCore groups to compare differentiation path differences. Finally, the dynamic expression patterns of cytotoxicity-related genes (GZMB, PRF1, IFNG), early differentiation-related genes (CCR7, IL7R, TCF7), and exhaustion-related genes (PDCD1, LAG3, HAVCR2) were visualized along the pseudotime axis, and a heatmap was generated to illustrate the temporal switching of functional modules.

### 2.15 Metabolic pathway and functional enrichment analyses at the single-cell level

To further characterize the functional states of key cell types within the tumor microenvironment, metabolic pathway activity was evaluated at the single-cell level using the scMetabolism package (version 0.2.1), a VISION-based framework for estimating pathway activity (63). This analysis quantified metabolic activity scores for 85 KEGG metabolic pathways across individual cells in the GSE132465 dataset, enabling systematic comparison of metabolic states among different cell types. In parallel, functional pathway enrichment analysis was conducted using the irGSEA package (version 3.3.2), which integrates multiple rank-based gene set scoring methods for single-cell transcriptomic data (64). Six built-in algorithms—AUCell, UCell, singscore, ssGSEA, JASMINE, and Viper—were applied via the irGSEA.score function to calculate pathway activity scores. Human Hallmark and KEGG gene sets were used as reference gene sets. Differences in pathway activity scores were assessed using the Wilcoxon test, with P < 0.05 considered statistically significant. To integrate enrichment results obtained from multiple scoring algorithms, the RobustRankAggreg package (version 1.2.1) (65) was employed, and the robust rank aggregation (RRA) method was applied. Gene sets showing significant enrichment (P < 0.05) across the majority of algorithms were retained for downstream interpretation of biological pathway characteristics associated with key cell types and other annotated cell populations.

### 2.16 Human tissue microarray and immunohistochemistry

A colorectal cancer tissue microarray containing 120 paired tumor and adjacent non-tumor tissues from the SYSUCC-CRC cohort was used for immunohistochemical validation. The use of the SYSUCC-CRC tissue microarray was approved by the Institutional Ethics Committee of Sun Yat-sen University Cancer Center (approval No. B2026-252-01). This retrospective study was conducted in accordance with the Declaration of Helsinki, and the requirement for informed consent was waived by the Ethics Committee. Tissue microarray slides were baked at 60 °C for 2h, deparaffinized in xylene, and rehydrated through a graded ethanol series. Antigen retrieval was performed in EDTA buffer (pH 9.0) using microwave heating. Endogenous peroxidase activity was blocked with 3% hydrogen peroxide for 15 min in the dark, followed by blocking with 5% bovine serum albumin for 30 min at room temperature. The sections were then incubated overnight at 4 °C with primary antibodies against NOX4 (ABclonal, China, Cat# A11274), CDKN2A (Proteintech, China, Cat# 10883-1-AP), CXCL1 (Proteintech, China, Cat# 12335-1-AP), and SIX1 (Proteintech, China, Cat# 10709-1-AP). After washing, the sections were incubated with the corresponding HRP-conjugated secondary antibodies for 1 h at room temperature, visualized using DAB, counterstained with hematoxylin, dehydrated, cleared, and mounted for microscopic examination and image acquisition.

Immunohistochemical staining was evaluated independently by two investigators blinded to the clinicopathological data. An IHC score was calculated by multiplying staining intensity (0, negative; 1, weak; 2, moderate; 3, strong) by the percentage of positively stained cells (0–100), yielding a final score ranging from 0 to 300. The mean score from the two observers was used for subsequent analysis. Clinicopathological characteristics of the SYSUCC-CRC tissue microarray cohort are summarized in **Supplementary Table 5**, and individual immunohistochemical scores for paired tumor and adjacent non-tumor tissues are provided in **Supplementary Table 6**.

### 2.17 Cell lines and cell culture

Human colorectal cancer cell lines (DLD1, SW620, SW480, HCT15, HCT116, HT29, and RKO) and the normal human intestinal epithelial cell line NCM460 were obtained from the American Type Culture Collection (ATCC; Manassas, VA, USA) and the Shanghai Cell Bank of the Chinese Academy of Sciences (Shanghai, China). All cell lines were cultured in RPMI 1640 medium (HyClone, Logan, UT, USA) supplemented with 10% fetal bovine serum (Invitrogen, Carlsbad, CA, USA) and maintained at 37 °C in a humidified incubator with 5% CO_2_.

### 2.18 Reverse transcription-quantitative PCR

Total RNA was extracted from NCM460 cells and CRC cell lines (DLD1, SW480, HCT15, HT29, and RKO) using TRIzol reagent according to the manufacturer’s instructions. Complementary DNA (cDNA) was synthesized from 1 µg of total RNA using the PrimeScript RT Master Mix Kit (Takara, Tokyo, Japan). RT-qPCR was subsequently performed using GoTaq qPCR Master Mix (Promega, Madison, WI, USA) following the manufacturer’s protocol. Gene expression levels were normalized to GAPDH expression. Detailed information on the RT-qPCR program parameters and primer sequences for prognostic genes is provided in **Supplementary Tables 2**, **S3**.

### 2.19 Cell transfection

SW480, SW620, HCT15 and HCT116 cells grown to 80%-90% confluence were taken out of the incubator. The old culture medium was discarded, and the cells were washed twice with 1× PBS. Subsequently, 1 mL of trypsin was added, and the cells were digested at 37 °C for 1–3 min. Then, 1 mL of complete medium was added to terminate the digestion. The cells were gently pipetted to obtain a single−cell suspension, and 10 µL of the suspension was taken for cell counting. A total of 2 mL of complete medium was added to each well of a 6−well plate, and 2 × 10^5^ cells per well were seeded. The plate was returned to the incubator and cultured for 24 h. When the cell density reached approximately 50% confluence, siNOX4 and negative control (siNC) transfection complexes were prepared in sterile EP tubes according to the manufacturer’s instructions of the transfection kit (Ribobio, China, Cat# R10043.9) and incubated at room temperature for 15 min. The transfection complexes were added dropwise to the corresponding wells. The plate was placed back into the 37 °C incubator and cultured for another 48 h.

### 2.20 Western blot

After transfection, SW480 and SW620 cells were collected, and total protein was extracted using RIPA lysis buffer. Protein concentrations were determined using a BCA kit. Equal amounts of protein samples were separated by SDS−PAGE (60 V for 30 min, followed by 100 V until the marker reached the bottom of the gel). PVDF membranes were activated by soaking in methanol for approximately 10 min, and then proteins were transferred at a constant voltage of 100 V for 90 min. After transfer, the membranes were blocked with a protein−free rapid blocking buffer (Pumoke, China, Cat# PMK007B) for 30 min at room temperature. The membranes were washed three times with PBST, each for 5 min. According to the molecular weights of the target proteins, the membranes were cut and incubated with primary antibodies against NOX4 (Yamei, China, Cat# R015010), PD−L1 (Yamei, China, Cat# R013735) and GAPDH (Proteintech, China, Cat# 10494−1−AP) overnight at 4 °C on a shaker. The next day, after washing, the membranes were incubated with the corresponding secondary antibody (Abbkine, China, Cat# A21020) for 1-2 h at room temperature. The secondary antibody was discarded, the developing solution was prepared, and the membranes were visualized using a chemiluminescence imaging system.

### 2.21 Quantitative real−time PCR for knockdown efficiency and immune−related gene expression

Forty−eight hours after transfection, SW480 and SW620 cells were collected, and total RNA was extracted using TRIzol reagent. cDNA was synthesized from 500 ng of total RNA using the SuperScript III First−Strand cDNA Synthesis System. qPCR was performed using TransStart Top Green qPCR SuperMix. The primer sequences were as follows: NOX4 forward CCAAGCAGGAGAACCAGGAG, reverse TCCCTTTTCTCCCCTCCCTC; IL−6 forward CTGCCGTCGTTTGGCTTTAC, reverse TTTCGGTGTTCTCCCTGGGT; CCL2 forward GACCATTGTGGCCAAGGAGA, reverse TTGGGTTTGCTTGTCCAGGT; TGF−β1 forward GCAGTGGCTGAACCAAGGAGAC, reverse GCCGTGAGCTGTGCAGGTG; MIF forward GCACAGCATCGGCAAGATC, reverse AGGCGAAGGTGGAGTTGTTC; PD−L1 forward TGCCGACTACAAGCGAATTACTG, reverse CTGCTTGTCCAGATGACTTCGG; PD−1 forward CTTCACCTGCAGCTTCTCCA, reverse GTTGGGCAGTTGTGTGACAC; GAPDH forward GAAGGTGAAGGTCGGAGTC, reverse GAAGATGGTGATGGGATTTC. All mRNA expression levels were calculated using the comparative Ct method, with GAPDH as the internal control.

### 2.22 Enzyme−linked immunosorbent assay

On the day after transfection, the cell culture supernatant from each well was collected and centrifuged at 1000×g for 20 min to remove debris and cell components. The supernatant was then collected. The secretion levels of IL−6 (Cat# ELK1156), MIF (Cat# ELK5252) and TGF−β1 (Cat# ELK10034) were measured using ELISA kits (ELK, China) according to the manufacturer’s instructions. A total of 100 µL of each sample was added to the bottom of the wells of the ELISA plate and incubated at 37 °C for 80 min. The liquid was discarded, 200 µL of wash buffer was added to each well, and the plate was incubated for 1-2 min before the buffer was discarded by patting the plate dry. Then, 100 µL of biotinylated antibody working solution was added to each well and incubated at 37 °C for 50 min, followed by three washes. Subsequently, 100 µL of enzyme conjugate working solution was added to each well, incubated at 37 °C for 50 min, and washed three times again. Then, 90 µL of TMB substrate solution was added to each well and incubated at 37 °C in the dark for 20 min. Finally, 50 µL of stop solution was added to terminate the reaction, and the optical density of each well was measured at 450 nm using a microplate reader.

### 2.23 Reactive oxygen species detection

The culture medium of transfected cells was discarded, and the cells were washed twice with PBS. DCFH−DA (Beyotime, China, Cat# S0033S) was diluted with PBS at a ratio of 1:1000 to a final concentration of 10 µM. The diluted DCFH−DA was added to cover the cells, and the cells were incubated at 37 °C in the cell culture incubator for 20 min. The cells were washed three times with PBS, and the ROS fluorescence intensity was detected using a fluorescence microscope or a fluorescence microplate reader.

### 2.24 Transwell migration assay

Forty−eight hours after transfection, when the cell confluence reached 80%-90%, the cells were trypsinized and collected. The cells were resuspended in complete medium, centrifuged at 1000 rpm for 5 min, the supernatant was discarded, and the cells were resuspended again and counted. A total of 2 × 10^5^ cells were seeded into the upper chamber of the Transwell insert (serum−free medium), and complete medium containing 15% fetal bovine serum (with double antibodies) was added to the lower chamber. The cells were cultured at 37 °C in a 5% CO_2_ incubator for 2 days. A clean cotton swab was used to gently remove the non−migrated cells from the upper chamber. The cells were fixed with 4% paraformaldehyde for 15 min and then stained with 0.1% crystal violet solution for 15 min at room temperature, followed by washing with DPBS. The migrated cells were observed and counted in five randomly selected fields under a light microscope for statistical analysis.

### 2.25 Statistical analysis

All statistical analyses were performed using R software (version 4.3.3). Comparisons between groups were conducted using the Wilcoxon test, unless otherwise specified. For RT-qPCR experiments, unpaired two-tailed Student’s t-tests were performed using GraphPad Prism software (version 8.0.2) to compare Ct values between groups. A P value < 0.05 was considered statistically significant.

## 3 Results

### 3.1 Identification and functional characterization of senescence and circadian rhythm-associated candidate genes in CRC

**Figure 1:**
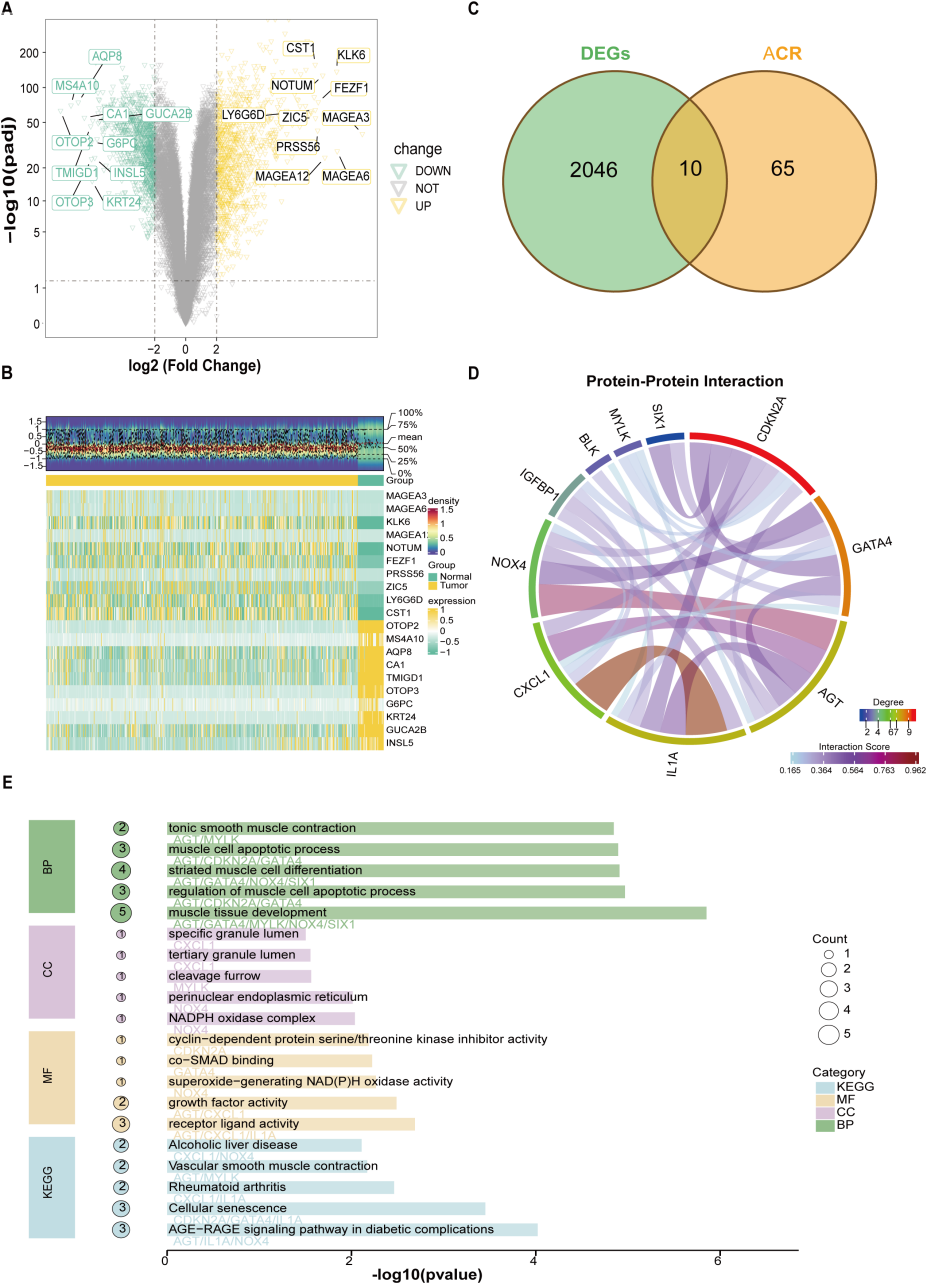
Identification and functional characterization of senescence- and circadian rhythm-related genes in colorectal cancer (CRC). **(A)** Volcano plot showing differentially expressed genes (DEGs) between CRC tumor tissues and adjacent non-tumor tissues in the training cohort. Upregulated genes are shown in yellow, downregulated genes in green, and non-significant genes in gray. **(B)** Heatmap illustrating the expression patterns of representative DEGs between CRC and normal samples. **(C)** Venn diagram depicting the intersection of DEGs, senescence-related genes, and circadian rhythm-related genes. ACR, Aging-Circadian Rhythm intersection **(D)** Gene Ontology (GO) enrichment analysis, including biological processes (BP), cellular components (CC), and molecular functions (MF), together with Kyoto Encyclopedia of Genes and Genomes (KEGG) pathway enrichment analysis of the 10 candidate genes. **(E)** Protein-protein interaction (PPI) network of the 10 candidate genes.

Differential expression analysis was performed in the training cohort by comparing CRC tumor tissues with adjacent non-tumor tissues. A total of 2,056 differentially expressed genes (DEGs) were identified, including 994 upregulated genes and 1,062 downregulated genes in CRC samples (**Figures 1A, B**). Intersecting these 2,056 DEGs with the 75 senescence-circadian associated genes (ACR genes) yielded 10 candidate genes (**Figure 1C**). Among them, AGT, CDKN2A, CXCL1, GATA4, IGFBP1, IL1A, NOX4, and SIX1 were upregulated in CRC, whereas BLK and MYLK were downregulated. Protein-protein interaction (PPI) network analysis showed that all 10 candidate genes were incorporated into a single interaction network, with no isolated nodes detected (**Figure 1D**). Functional enrichment analysis demonstrated that these genes were significantly enriched (P < 0.05) in 595 Gene Ontology (GO) terms, including 552 biological processes (BP), 12 cellular components (CC), and 31 molecular functions (MF) (**Figure 1E**; **Supplementary Table 4**). At the biological process level, enriched terms included tonic smooth muscle contraction and regulation of muscle cell apoptotic processes. At the cellular component level, the candidate genes were enriched in the perinuclear endoplasmic reticulum and apical plasma membrane. At the molecular function level, enrichment was observed in cytokine activity, growth factor receptor binding, and receptor ligand activity. KEGG analysis identified 12 significantly enriched pathways, among which cellular senescence was notable (**Figure 1E**; **Supplementary Table 4**). Overall, these enrichments suggested that the candidate genes were mainly involved in stress-regulatory and microenvironment-related processes within the senescence-circadian overlap.

### 3.2 Development and validation of an RSF-derived Senescence-Circadian Interplay Score for CRC prognostic stratification

**Figure 2:**
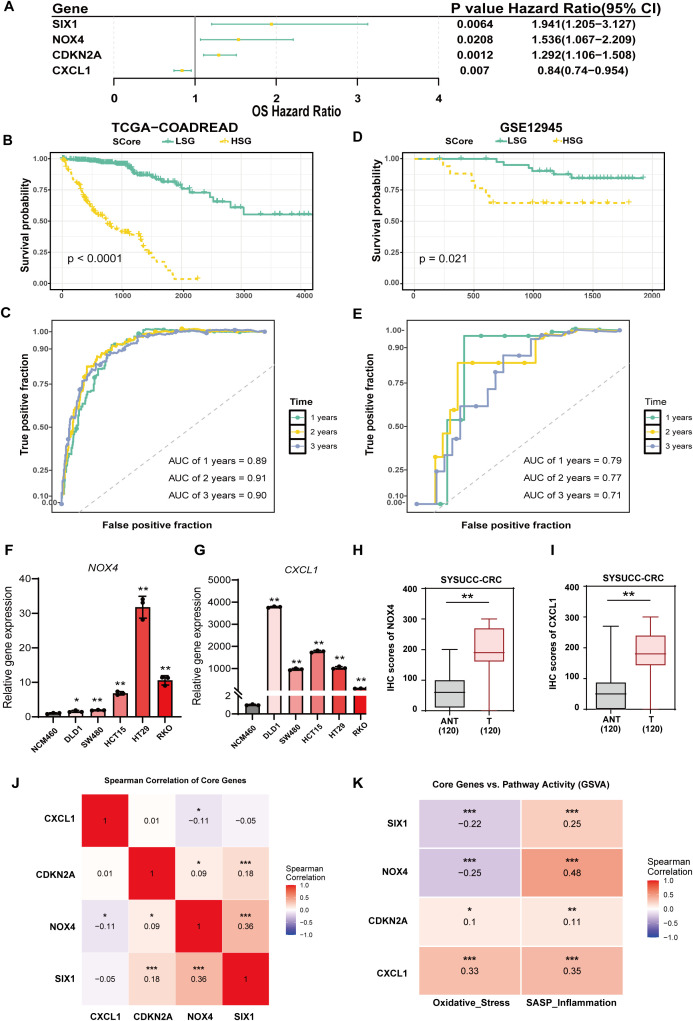
Development, validation, and experimental verification of a senescence- and circadian rhythm-related prognostic risk model in CRC. **(A)** Forest plot of univariate Cox proportional hazards regression analysis showing the associations between NOX4, CXCL1, CDKN2A, and SIX1 expression and overall survival in the training cohort. **(B)** Kaplan-Meier survival curves comparing overall survival between the high-SCore group (HSG) and low-SCore group (LSG) in the training cohort. **(C)** Time-dependent receiver operating characteristic (ROC) curves evaluating the predictive performance of the risk model in the training cohort, with area under the curve (AUC) values shown for 1-, 2-, and 3-year overall survival. **(D)** Kaplan-Meier survival curves comparing overall survival between the HSG and LSG in the external validation cohort GSE12945. **(E)** Time-dependent ROC curves assessing the predictive accuracy of the prognostic model in GSE12945, with AUC values shown for 1-, 2-, and 3-year overall survival. **(F, G)** Relative mRNA expression levels of the four prognostic genes in the normal colon epithelial cell line NCM460 and CRC cell lines DLD1, SW480, HCT15, HT29, and RKO, as measured by reverse transcription-quantitative PCR (RT-qPCR): NOX4 **(F)**, CXCL1 **(G)**. **(H, I)** Quantitative immunohistochemical validation of NOX4 and CXCL1 expression in the SYSUCC-CRC tissue microarray. IHC scores were significantly higher in colorectal cancer tissues than in paired adjacent non-tumor tissues (n = 120). **(J)** Spearman correlation heatmap among prognostic genes. The heatmap displays pairwise expression correlations among the four prognostic genes (CXCL1, CDKN2A, NOX4, SIX1). Color gradient represents the magnitude of Spearman correlation coefficients, with red indicating positive correlation and blue indicating negative correlation. Numerical values within cells indicate correlation coefficients. Significance annotation: *P < 0.05, **P < 0.01, ***P < 0.001. All P values were adjusted by the Benjamini−Hochberg (BH) method for multiple testing. **(K)** Spearman correlation heatmap between prognostic gene expression and pathway activity. The heatmap displays correlations between the expression levels of the four prognostic genes and the GSVA activity scores of the oxidative stress and SASP inflammatory response pathways. Color gradient represents the magnitude of Spearman correlation coefficients, with red indicating positive correlation and blue indicating negative correlation. Numerical values within cells indicate correlation coefficients. Significance annotation: *P < 0.05, **P < 0.01, ***P < 0.001. All P values were adjusted by the BH method for multiple testing.

Univariate Cox proportional hazards regression analysis was performed in 524 CRC patients with complete survival information from the training cohort. Four genes—CXCL1, CDKN2A, NOX4, and SIX1—were identified as significantly associated with overall survival (P < 0.05) (**Figure 2A**). Among these genes, CDKN2A, NOX4, and SIX1 exhibited hazard ratios (HRs) greater than 1, whereas CXCL1 showed an HR less than 1. All four genes satisfied the proportional hazards assumption (P > 0.05) (**Supplementary Figures 2A–D**). An RSF approach was then used to derive a continuous risk score (SCore) that integrates nonlinear effects and interactions among the retained predictors. Using an optimal cutoff value of 29.6248, patients in the training cohort (n = 524) were stratified into a high-SCore group (HSG; n = 168) and a low-SCore group (LSG; n = 356). An increasing SCore was accompanied by a higher frequency of mortality events (**Supplementary Figure 2E**). Kaplan-Meier survival analysis showed that overall survival was significantly lower in the HSG compared with the LSG (P < 0.0001) (**Figure 2B**). Time-dependent receiver operating characteristic (ROC) analysis demonstrated strong predictive performance of the model in the training cohort, with area under the curve (AUC) values of 0.89, 0.91, and 0.90 for 1-, 2-, and 3-year survival, respectively (**Figure 2C**). The prognostic model was further validated in the external GSE12945 cohort. Using an optimal cutoff value of 32.40562, 62 CRC samples were stratified into the HSG (n = 17) and LSG (n = 45). Survival analyses and predictive performance in the validation cohort mirrored those observed in the training cohort (**Supplementary Figure 2F**, **Figures 2D, E**). As supplementary cross-cohort support, analysis of the larger GSE39582 dataset (n = 556) also showed significantly worse overall survival in patients with higher SCore values than in those with lower SCore values (P < 0.0001; **Supplementary Figures 3A, B**), although the time-dependent ROC performance in this cohort was modest (**Supplementary Figure 3C**).

To experimentally assess baseline expression of the four prognostic genes, RT-qPCR was performed in the normal colon epithelial cell line NCM460 and five CRC cell lines (DLD1, SW480, HCT15, HT29, and RKO). NOX4 expression was significantly increased in SW480, HCT15, HT29, and RKO cells (P < 0.05), while no significant difference was observed in DLD1 cells (**Figure 2F**). CXCL1 expression was significantly upregulated in all CRC cell lines compared with NCM460, with the highest expression observed in DLD1 cells (P < 0.01) (**Figure 2G**). In contrast, CDKN2A and SIX1 showed non-uniform expression patterns across CRC cell lines, indicating marked inter-cell-line heterogeneity (**Supplementary Figures 4A, B**). CDKN2A was significantly downregulated in DLD1 cells but upregulated in HCT15, HT29, and RKO cells (P < 0.05). SIX1 was significantly downregulated in DLD1, SW480, HCT15, and RKO cells compared with NCM460 (P < 0.05), whereas no significant difference was detected in HT29 cells.

To further validate tissue-level expression of the four prognostic genes, immunohistochemical analysis was performed using the SYSUCC-CRC tissue microarray containing 120 paired colorectal cancer and adjacent non-tumor tissues. NOX4 and CXCL1 showed significantly higher IHC scores in CRC tissues than in paired adjacent non-tumor tissues (**Figures 2H, I**). Consistently, CDKN2A and SIX1 were also expressed at significantly higher levels in CRC tissues than in paired adjacent non-tumor tissues (**Supplementary Figures 4C, D**). Representative IHC staining images further illustrated stronger staining intensity in tumor tissues relative to adjacent non-tumor tissues (**Supplementary Figure 4E**). Detailed clinicopathological characteristics of the tissue microarray cohort and case-level IHC scores are provided in **Supplementary Tables 5**, **S6**, respectively. Together, these results provide tissue-level support for aberrant expression of the four prognostic proteins in CRC and complement the transcriptomic and cell-line-based validation results.

### 3.3 Prognostic genes are embedded in a shared oxidative stress- and inflammatory-related transcriptional context

To explore the transcriptional regulatory relationships between the four prognostic genes (CXCL1, CDKN2A, NOX4, SIX1) and the oxidative stress and inflammatory response pathways, co−expression and pathway activity analyses were further performed in this study. Spearman correlation analysis showed significant positive correlations among the four prognostic genes after BH correction (P < 0.05), with relatively higher correlation coefficients observed between NOX4 and CDKN2A, as well as between CXCL1 and SIX1, suggesting possible synergistic regulation at the transcriptional level (**Figure 2J**). Using a significance threshold of |rho| ≥ 0.3 and FDR < 0.05 for co−expression, the numbers of significantly co−expressed genes between each core gene and the oxidative stress or inflammatory response pathway were analyzed. The results indicated that NOX4 exhibited the highest number of co−expressed genes with the oxidative stress pathway, while CXCL1 showed the highest number with the inflammatory response pathway. CDKN2A and SIX1 also displayed moderate numbers of significantly co−expressed genes in both pathways (**Supplementary Figure 4F**). Furthermore, pathway activity scores were calculated by GSVA and correlated with the expression of the core genes. The correlation analysis demonstrated that the expression levels of NOX4 and CXCL1 were significantly positively correlated with the activity of the oxidative stress pathway (BH−adjusted P < 0.05). Similarly, CXCL1 and SIX1 were significantly positively correlated with the activity of the inflammatory response pathway, whereas CDKN2A showed a moderately positive correlation with the activity scores of both pathways (**Figure 2K**). Together, these findings support the coexistence of the four prognostic genes within a coordinated oxidative stress- and inflammation-related transcriptional context in CRC.

### 3.4 Clinical evaluation of the SCore and development of a prognostic nomogram in CRC

**Figure 3:**
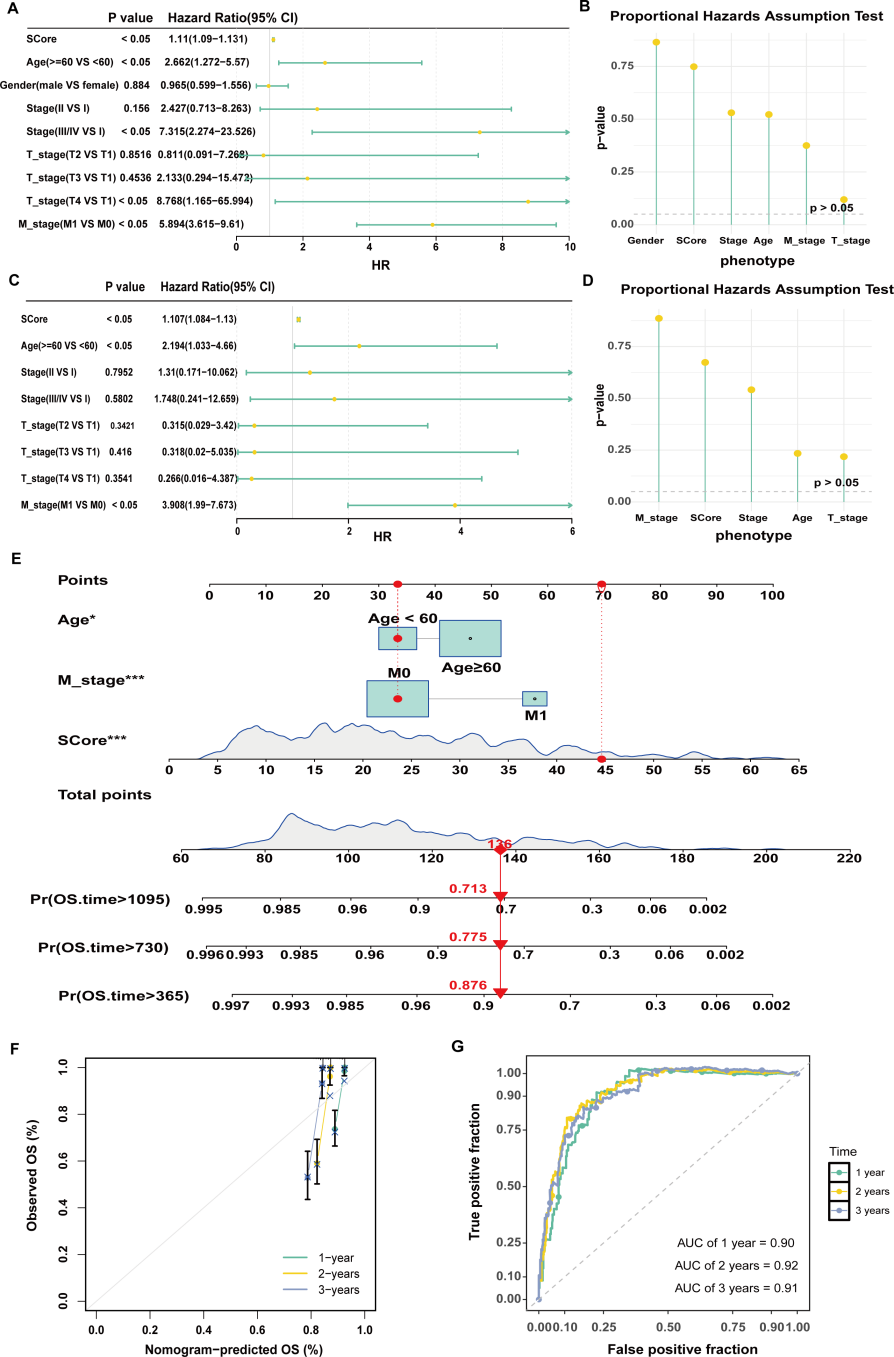
Prognostic evaluation of the SCore and construction of a nomogram in CRC. **(A)** Forest plot of univariate Cox regression analysis evaluating the prognostic significance of the SCore and clinicopathological variables, including age, sex, T stage, M stage, and pathological stage. **(B)** Proportional hazards (PH) assumption test based on Schoenfeld residuals for variables included in the univariate Cox regression model. **(C)** Forest plot of multivariate Cox regression analysis identifying independent prognostic factors for overall survival in CRC. **(D)** Schoenfeld residual-based PH assumption test for variables included in the multivariate Cox regression model. **(E)** Nomogram integrating independent prognostic factors (SCore, age, and M stage) to predict 1-, 2-, and 3-year overall survival probabilities in CRC patients. **(F)** Calibration curves assessing the agreement between predicted and observed 1-, 2-, and 3-year overall survival probabilities. **(G)** Time-dependent ROC curves assessing the discriminative performance of the nomogram for 1-, 2-, and 3-year overall survival. *P < 0.05; ***P < 0.001.

Associations between clinicopathological characteristics and SCore were evaluated in the training cohort. SCore showed significant differences across M stage, T stage, and overall pathological stage, with progressively higher SCore observed in more advanced stages (P < 0.05) (**Supplementary Figures 5A–C**). In contrast, no significant differences in SCore were observed with respect to age or sex (**Supplementary Figures 5D, E**). The prognostic value of the SCore and clinicopathological variables was further assessed using Cox regression analyses. Univariate Cox regression identified the SCore, age, M stage, T stage, and overall stage as variables significantly associated with overall survival (HR ≠ 1, P < 0.05) (**Figure 3A**). All variables satisfied the proportional hazards assumption (P > 0.05) (**Figure 3B**). Multivariate Cox regression analysis demonstrated that the SCore, age, and M stage remained significantly associated with overall survival (HR ≠ 1, P < 0.05) (**Figure 3C**), and these variables also passed the proportional hazards assumption test (P > 0.05) (**Figure 3D**). Based on these independent prognostic variables, a nomogram was constructed to estimate 1-, 2-, and 3-year overall survival probabilities for patients with CRC (**Figure 3E**). Calibration curves showed good agreement between predicted and observed survival probabilities (**Figure 3F**). Time-dependent ROC analysis demonstrated that the nomogram achieved AUC values exceeding 0.9 at 1, 2, and 3 years (**Figure 3G**).

We further compared the detailed clinicopathological characteristics between the high-SCore group (HSG) and the low-SCore group (LSG) (**Table 1**). The results showed a significant difference in sex distribution between the two groups (P = 0.039), with a higher proportion of male patients in the LSG (59.3% vs. 41.8%). Regarding T stage and M stage, the proportions of patients with T4 (18.2% vs. 4.8%) and M1 (27.3% vs. 8.3%) were significantly higher in the HSG than in the LSG (P = 0.009 and P = 0.001, respectively). Overall pathological stage also demonstrated a higher proportion of stage III/IV patients in the HSG (50.9% vs. 33.1%, P = 0.048). However, no statistically significant differences were observed between the two groups in MSI status, tumor location, histological type, or age group (P > 0.05). These results indicate that the high-risk state defined by the SCore may be associated with clinical features related to tumor invasion and metastasis (T stage, M stage, and overall stage).

**Table 1:** Baseline clinicopathological characteristics of colorectal cancer patients stratified by senescence–circadian interplay score (SCore) in the subset with complete clinicopathological annotations.

Characteristic	Level	HSG (n = 55)	LSG (n = 145)	P value
N		55	145	
MSI status (%)	MSI-H	8 (14.5)	23 (15.9)	0.385
	MSI-L	12 (21.8)	20 (13.8)	
	MSS	35 (63.6)	102 (70.3)	
Tumor site (%)	Left	13 (23.6)	48 (33.1)	0.341
	Rectum	19 (34.5)	36 (24.8)	
	Right	21 (38.2)	51 (35.2)	
	Transverse	2 (3.6)	10 (6.9)	
Histological type (%)	Colon Adenocarcinoma	29 (52.7)	98 (67.6)	0.23
	Colon Mucinous Adenocarcinoma	7 (12.7)	12 (8.3)	
	Rectal Adenocarcinoma	17 (30.9)	29 (20.0)	
	Rectal Mucinous Adenocarcinoma	2 (3.6)	6 (4.1)	
Sex (%)	Female	32 (58.2)	59 (40.7)	0.039
	Male	23 (41.8)	86 (59.3)	
Age (%)	Age < 60	11 (20.0)	31 (21.4)	0.984
	Age ≥ 60	44 (80.0)	114 (78.6)	
Pathological stage (%)	Stage I	8 (14.5)	38 (26.2)	0.048
	Stage II	19 (34.5)	59 (40.7)	
	Stage III/IV	28 (50.9)	48 (33.1)	
T stage (%)	T1	2 (3.6)	5 (3.4)	0.009
	T2	7 (12.7)	38 (26.2)	
	T3	36 (65.5)	95 (65.5)	
	T4	10 (18.2)	7 (4.8)	
M stage (%)	M0	40 (72.7)	133 (91.7)	0.001
	M1	15 (27.3)	12 (8.3)	

### 3.5 Somatic mutation characteristics and pathway enrichment in high- and low-SCore groups

**Figure 4:**
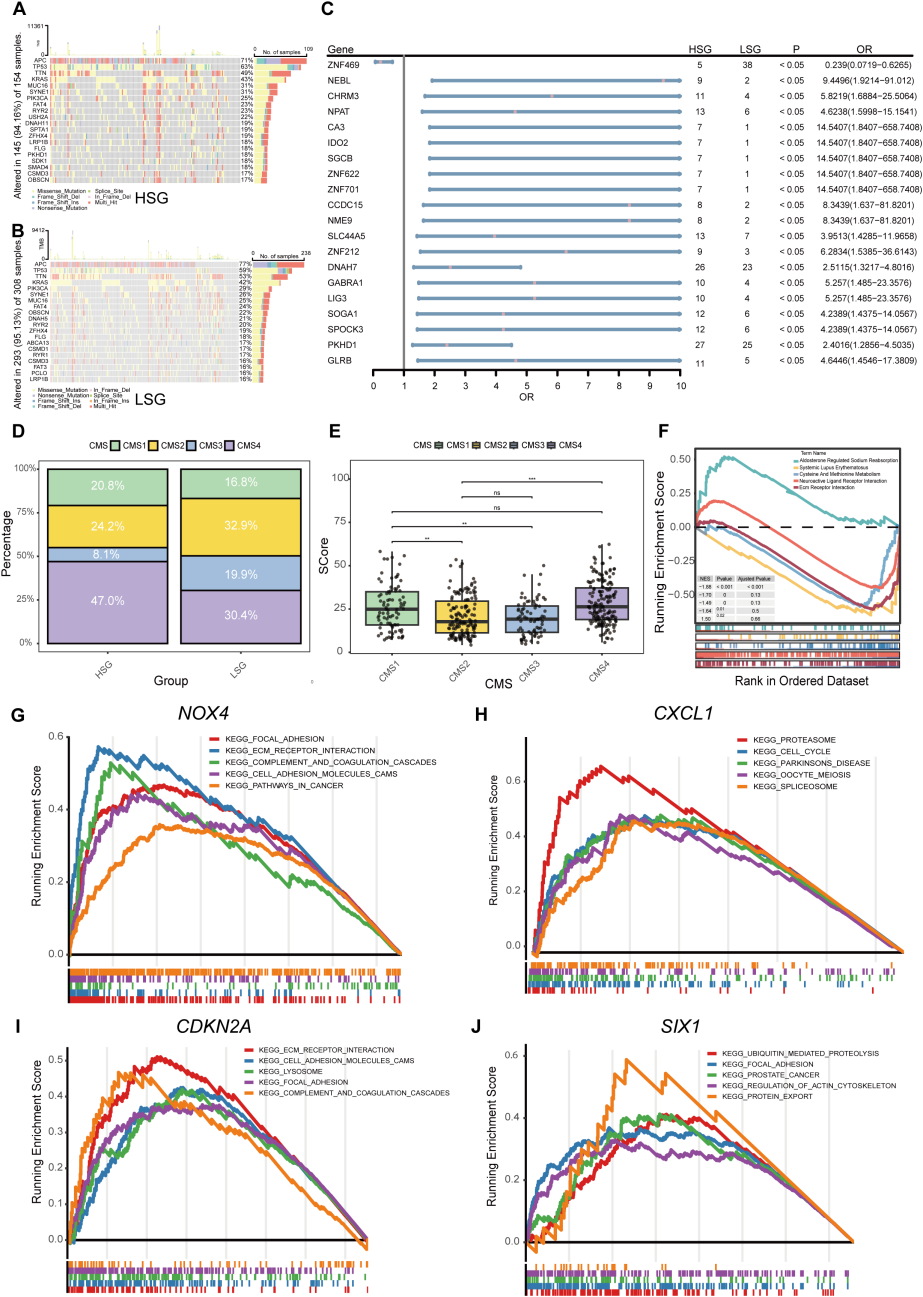
Somatic mutation landscape and pathway enrichment analyses in high- and low-SCore CRC groups. **(A)** Oncoplot showing the somatic mutation landscape of CRC patients in the high-SCore group (HSG). Each column represents one patient and each row represents a gene; colors indicate mutation types. **(B)** Oncoplot depicting the somatic mutation profile of CRC patients in the low-SCore group (LSG). **(C)** Forest plot comparing mutation frequencies of differentially mutated genes between the HSG and LSG. **(D)** Distribution of consensus molecular subtypes (CMS1-CMS4) between the HSG and LSG. **(E)** Comparison of SCore distributions among CMS subtypes, *P < 0.05, **P < 0.01, ***P < 0.001, ns represents not significant. **(F)** Gene set enrichment analysis (GSEA) showing commonly enriched pathways in the HSG and LSG. **(G-J)** GSEA enrichment plots illustrating the top enriched pathways associated with NOX4 **(G)**, CXCL1 **(H)**, CDKN2A **(I)**, and SIX1 **(J)**.

Somatic mutation profiles were analyzed in patients from the high-SCore group (HSG) and low-SCore group (LSG). Somatic mutations were detected in 145 samples (94.16%) in the HSG and 293 samples (95.13%) in the LSG (**Figures 4A, B**). APC was the most frequently mutated gene in both groups, followed by TP53 and TTN. Missense mutations represented the predominant mutation type in both HSG and LSG. Correlation analysis showed that several genes, including NPAT and SPOCK3, were positively associated with high-SCore status (**Figure 4C**). Consensus molecular subtype (CMS) classification was successfully assigned to 472 CRC samples, including 85 CMS1, 143 CMS2, 76 CMS3, and 168 CMS4 cases. CMS4 represented the largest proportion in both HSG and LSG (**Figure 4D**). SCore differed significantly among CMS subtypes, with higher SCore observed in CMS1 and CMS4 compared with CMS2 and CMS3 (P < 0.01) (**Figure 4E**). Gene set enrichment analysis identified nine pathways enriched in both HSG and LSG (**Figure 4F**; **Supplementary Table 7**). The aldosterone-regulated sodium reabsorption pathway showed significant enrichment in the HSG (NES > 1, P < 0.05). In addition, GSEA revealed that SIX1, NOX4, CDKN2A, and CXCL1 were associated with 31, 48, 24, and 71 enriched pathways, respectively (**Supplementary Table 7**). Among the top enriched pathways, CDKN2A-, NOX4-, and SIX1-associated gene sets consistently showed enrichment in focal adhesion (**Figures 4G, I, J**). In addition, NOX4 and CDKN2A were enriched in extracellular matrix (ECM)-receptor interaction, complement and coagulation cascades, and cell adhesion molecules (CAMs) (**Figures 4G, I**). By contrast, CXCL1-associated genes were predominantly enriched in cell cycle- and proteasome-related pathways (**Figure 4H**).

### 3.6 Integrated analysis of immune landscape, immune evasion, and drug sensitivity in CRC SCore groups

**Figure 5:**
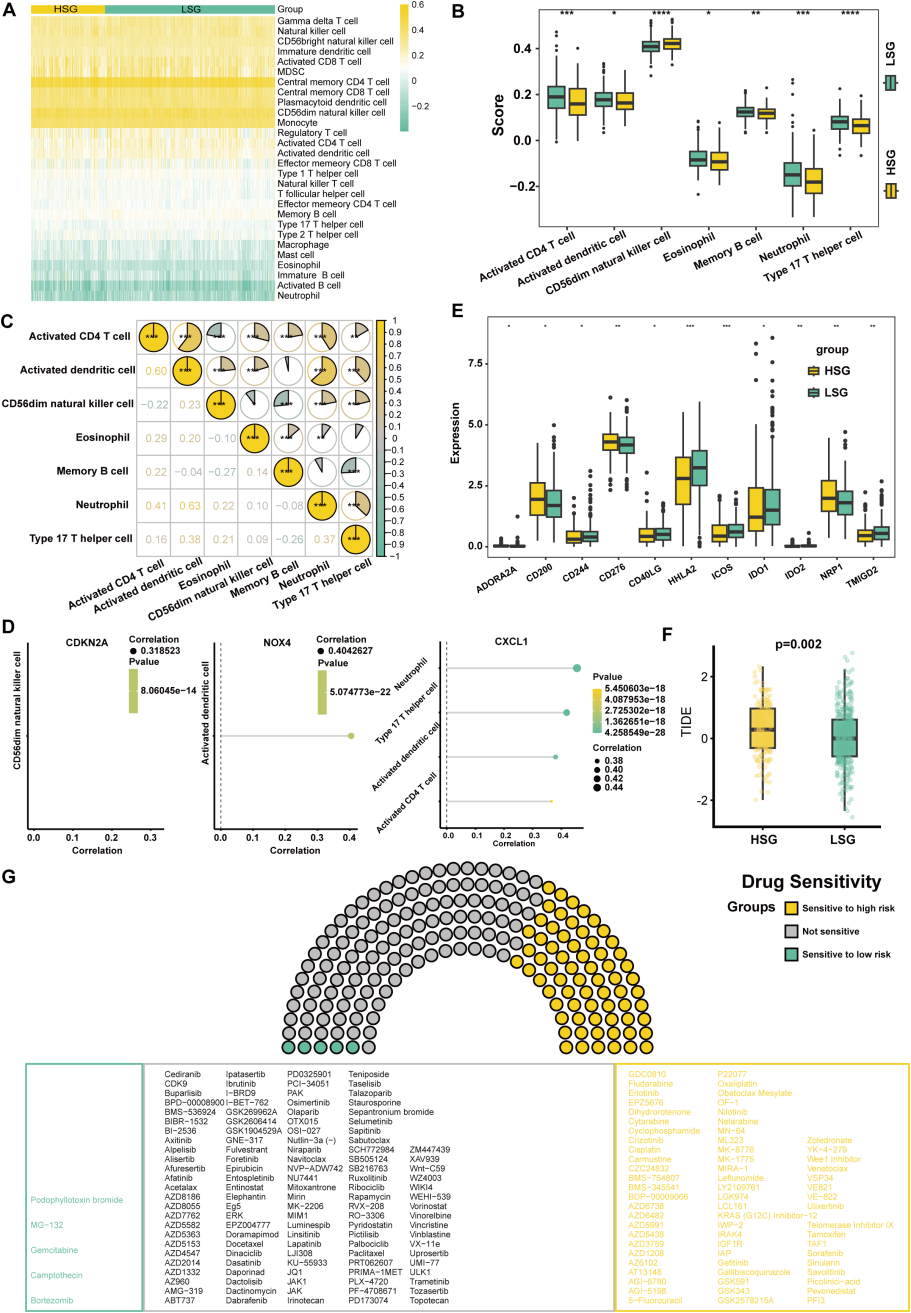
Immune landscape, immune evasion features, and drug sensitivity analyses in CRC. **(A)** Heatmap showing the relative infiltration levels of 28 immune cell types in the HSG and LSG, estimated using single-sample gene set enrichment analysis (ssGSEA). **(B)** Boxplots comparing infiltration scores of seven immune cell types. *P < 0.05, **P < 0.01, ***P < 0.001, ****P < 0.0001. **(C)** Correlation matrix depicting relationships among differentially infiltrated immune cell types. Circle size and color indicate correlation strength and direction. *P < 0.05, **P < 0.01, ***P < 0.001. **(D)** Correlation analysis between prognostic gene expression and differentially infiltrated immune cell types. **(E)** Differential expression of immune checkpoint-related genes between the HSG and LSG. *P < 0.05, **P < 0.01, ***P < 0.001. **(F)** Boxplot comparing Tumor Immune Dysfunction and Exclusion (TIDE) scores between the HSG and LSG. *P < 0.05, **P < 0.01. **(G)** Drug sensitivity analysis showing differences in predicted half-maximal inhibitory concentration (IC_50_) values between the HSG and LSG.

The infiltration levels of 28 immune cell types were evaluated in the HSG and LSG, revealing distinct immune infiltration patterns between the two groups (**Figure 5A**). Seven immune cell types exhibited significant differences in infiltration levels between HSG and LSG (**Figure 5B**). Correlation analysis indicated that neutrophils and activated dendritic cells showed the strongest positive correlation (cor = 0.63, P < 0.001) (**Figure 5C**). Associations between prognostic gene expression and immune cell infiltration were further assessed. NOX4 expression showed a positive correlation with activated dendritic cells (cor = 0.40, P < 0.001) (**Figure 5D**). CXCL1 expression was positively correlated with neutrophils (cor = 0.44, P < 0.001), while CDKN2A expression was positively correlated with CD56dim natural killer cells (cor = 0.32, P < 0.001). Expression analysis of immune checkpoint-related genes revealed that 11 checkpoint genes, including CD276, IDO1, and CD200, were differentially expressed between HSG and LSG (P3< 0.05) (**Figure 5E**). TIDE analysis demonstrated that TIDE scores were significantly higher in the HSG compared with the LSG (**Figure 5F**). Drug sensitivity analysis identified 73 drugs with significantly different predicted IC_50_ values between the two groups (P < 0.05), of which 69 drugs exhibited higher IC_50_ values in the HSG and 4 drugs exhibited higher IC_50_ values in the LSG (**Figure 5G**).

### 3.7 Cellular heterogeneity analysis identifies T cells as a key SCore-associated cell type in CRC

**Figure 6:**
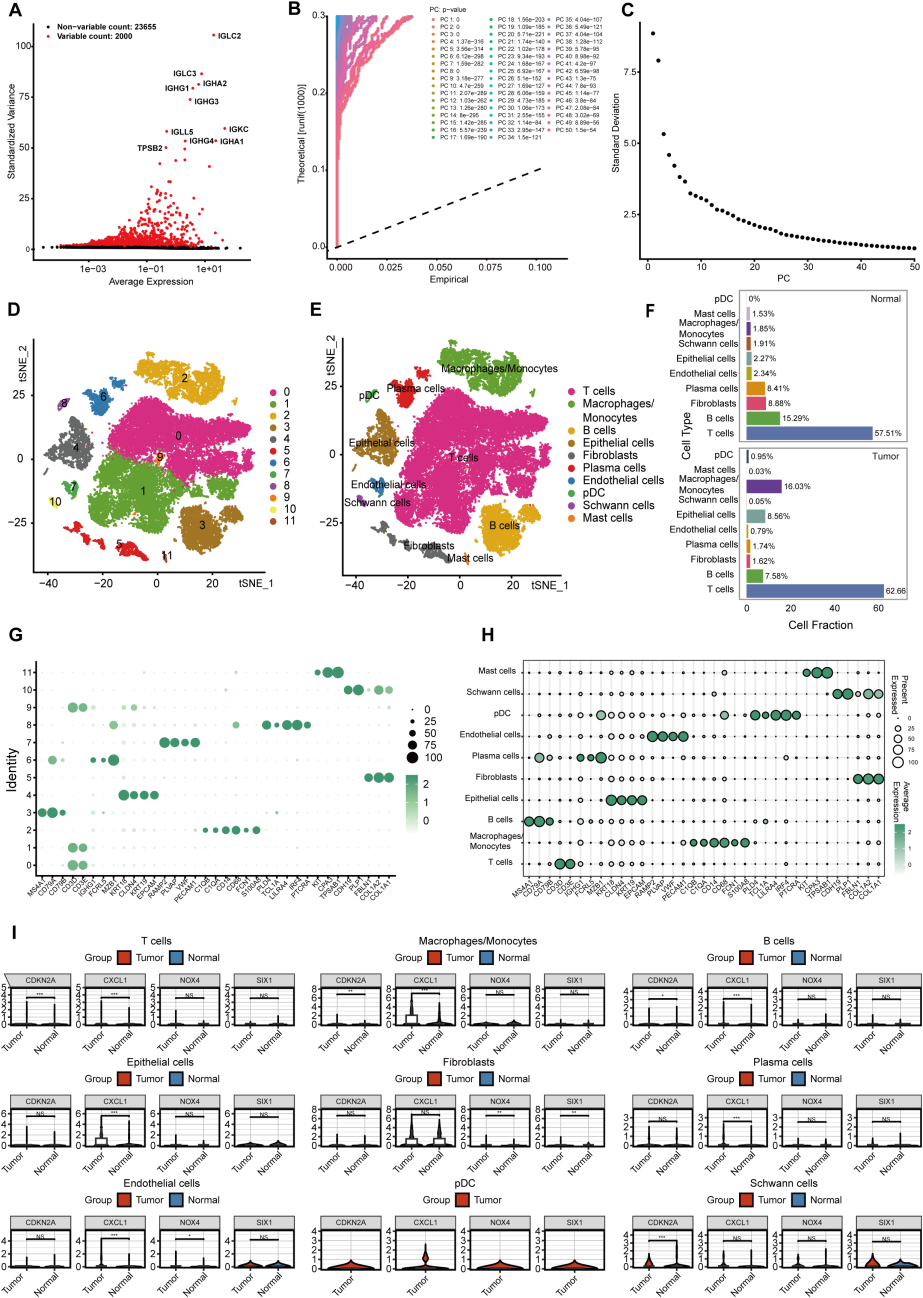
Single-cell transcriptomic analysis in CRC. **(A)** Identification of highly variable genes (HVGs) in the GSE132465 single-cell dataset. **(B)** Principal component analysis (PCA) significance testing using JackStraw analysis. **(C)** PCA scree plot showing the variance explained by each principal component. **(D)** t-distributed stochastic neighbor embedding (t-SNE) plot showing clustering of single cells based on selected principal components. **(E)** Dot plot displaying canonical marker gene expression across the 12 clusters for cell type annotation. **(F)** Dot plot summarizing marker gene expression across annotated major cell types. **(G)** Annotated t-SNE diagram, with different colors representing different clusters. **(H)** Bar plot comparing the proportions of different cell types between CRC tumor and normal samples. **(I)** Violin plots showing expression levels of CDKN2A, CXCL1, NOX4, and SIX1 across major cell types in tumor and normal samples, *P < 0.05, **P < 0.01, ***P < 0.001, NS represents not significant.

After quality control of the GSE132465 dataset, 36,625 high-quality cells were retained from an initial total of 63,689 cells, with 25,655 genes detected (**Supplementary Figures 6A, B**). A total of 2,000 highly variable genes were identified (**Figure 6A**), and the top 30 principal components with P < 0.05 were selected for downstream analyses (**Figures 6B, C**). Unsupervised clustering based on t-SNE dimensionality reduction identified 12 cell clusters, which were annotated into 10 major cell types according to marker gene expression, including B cells, macrophages/monocytes, T cells, epithelial cells, fibroblasts, plasma cells, endothelial cells, plasmacytoid dendritic cells, Schwann cells, and mast cells (**Figures 6D–G**; **Table 2**). T cells constituted a major immune compartment in both tumor and adjacent non-tumor tissues (**Figure 6H**). They were prioritized for downstream analysis not merely because of abundance, but because multiple SCore-associated features converged most clearly in this compartment. In particular, CDKN2A and CXCL1 showed significant tumor-versus-normal differences within T cells (P < 0.001) (**Figure 6I**), and subsequent cell–cell communication and pseudotime analyses further localized prominent risk-associated interactions and dynamic gene-expression changes to this population. Together, these findings supported the prioritization of T cells for follow-up analysis.

**Table 2:** Marker genes used for annotation of major cell types identified in the single-cell RNA sequencing analysis.

Cell types	Marker genes	Clusters
B cells	MS4A1, CD79A, CD79B	3
T cells	CD3D, CD3E	0、1、9
Plasma cells	IGHG1, FCRL5, MZB1	6
Epithelial cells	KRT18, CLDN4, KRT19, EPCAM	4
Endothelial cells	RAMP2, PLVAP, VWF, PECAM1	7
Macrophages	C1QB, C1QA, CD14, CD68	2
Monocytes	FCN1, S100A8	2
Plasmacytoid dendritic cells (pDCs)	PLD4, TCL1A, LILRA4, IRF4, PTCRA	8

We next sought to resolve T-cell heterogeneity at higher resolution and to localize the expression patterns of the four SCore genes within specific T-cell states. To this end, all T cells were independently re-clustered, yielding seven T-cell subpopulations with distinct molecular features (**Supplementary Figures 7A, B**). Functional annotation based on canonical markers identified cluster 0 as CD4+ conventional T cells (CD4, IL7R), clusters 1 and 3 as CD8+ cytotoxic T cells (CD8A, GZMB), cluster 2 as regulatory T cells (FOXP3, IL2RA), cluster 4 as naive T cells (CCR7, SELL), cluster 5 as proliferating T cells (MKI67, TOP2A), and cluster 6 as memory T cells (S100A4, CD44) (**Supplementary Figures 7C, D**).

We then examined the distribution of the four SCore genes across these T-cell subpopulations. Distinct subset-associated expression patterns were observed (**Supplementary Figure 7E**): CXCL1 was relatively enriched in proliferating and memory T cells, CDKN2A was most prominent in proliferating T cells, NOX4 showed preferential expression in Tregs, and SIX1 was more highly expressed in CD8+ cytotoxic T cells. These findings indicate that the four SCore genes are not uniformly expressed across the T-cell compartment, but instead localize to different functional T-cell subsets, suggesting its potential involvement in immune remodeling heterogeneity through effects on T-cell functional states.

To further characterize the T-cell states associated with SCore, we performed a focused analysis within the T-cell compartment (n = 22,413). T cells were divided into high- and low-SCore groups according to the median SCore. Differential analysis showed significant differences in exhaustion-related markers (PDCD1, LAG3, HAVCR2, and CTLA4) as well as the senescence-associated marker CDKN2A between the two groups. Violin plots and dot plots indicated that these differences were mainly attributable to changes in the proportion of expressing cells within specific T-cell subsets and to shifts in the high-expression tail, rather than to large differences in overall median expression (**Supplementary Figures 8A, B**). t-SNE feature plots further showed that SCore was continuously distributed across the T-cell compartment rather than confined to a discrete subset, consistent with a functional-state gradient (**Supplementary Figure 8C**).

Pseudotime analysis further revealed dynamic changes in SCore along T-cell differentiation trajectories. SCore showed an overall pattern of initial decline followed by a later increase along pseudotime (**Supplementary Figures 9A, B**). When trajectories were reconstructed separately for the high- and low-SCore groups, both groups formed multi-state differentiation networks, but their trajectory geometries and terminal branch orientations differed (**Supplementary Figure 9C**), indicating that distinct SCore states were associated with different patterns of T-cell state organization. Along pseudotime, cytotoxicity-related genes (GZMB, PRF1, and IFNG) and exhaustion-related genes (PDCD1, LAG3, and HAVCR2) gradually increased at later stages, whereas early differentiation-associated genes (CCR7, IL7R, and TCF7) were relatively higher at earlier stages and then declined (**Supplementary Figure 9D**). The pseudotime heatmap further supported a temporal transition from an early differentiation program to an intermediate transition state and then to a more pronounced late effector/exhaustion-associated program. Collectively, these findings support that SCore is associated with the dynamic evolution of T-cell functional states, with higher SCore states showing closer alignment with later stress-associated and exhaustion-related features.

### 3.8 Integrated communication, trajectory, and metabolic-functional context of T cells in CRC

**Figure 7:**
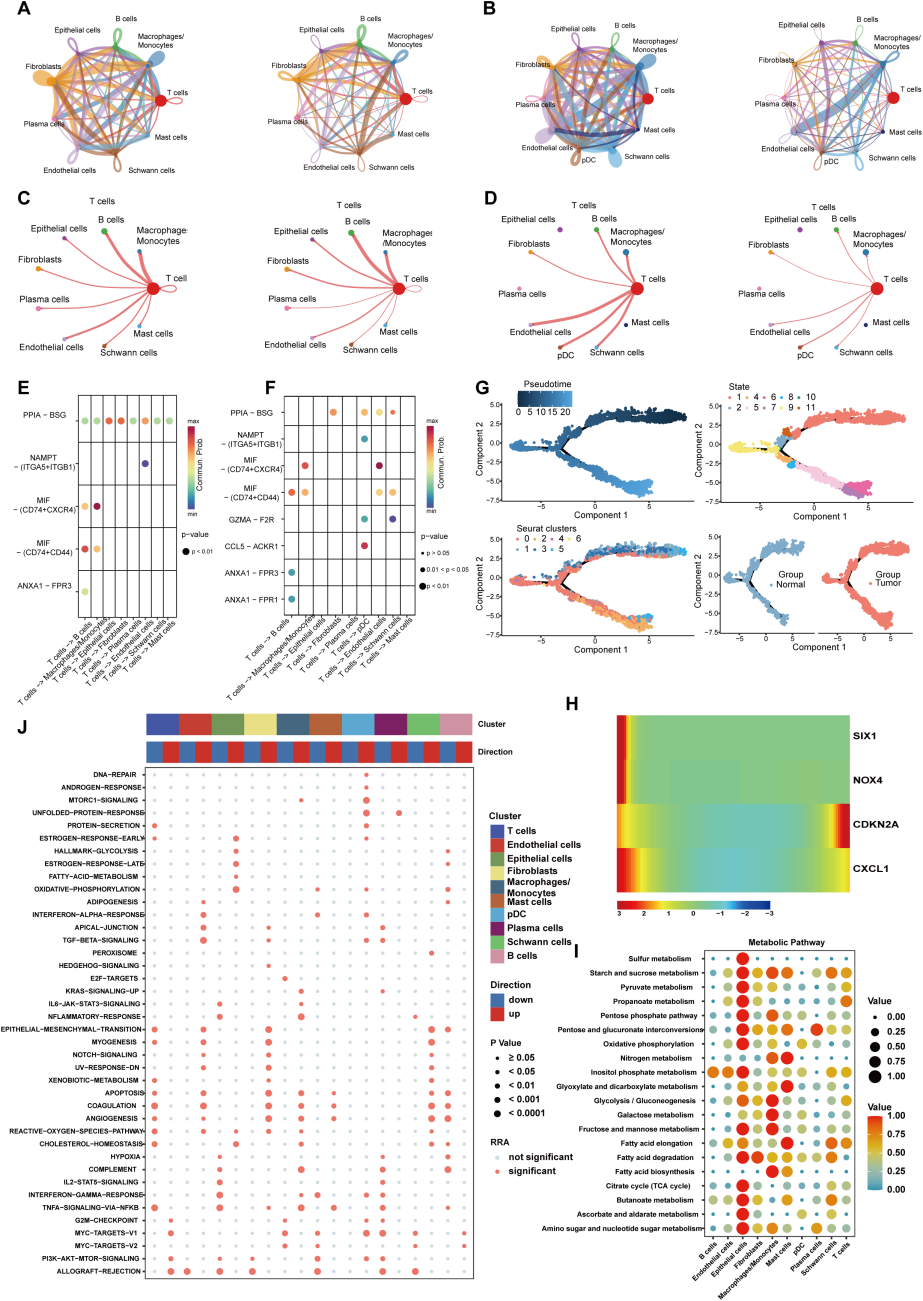
Cell-cell communication, pseudotime dynamics, and functional characteristics of T cells in CRC. **(A, B)** Global cell-cell communication networks inferred from ligand-receptor interactions in normal colorectal tissues **(A)** and CRC tumor tissues **(B)**. Node size indicates the number of interactions, and edge thickness reflects interaction strength. **(C, D)** T cell-centered communication networks in normal tissues **(C)** and CRC tumor tissues **(D)**. **(E,3F)** Bubble plots showing ligand-receptor interactions between T cells and other cell types in normal tissues **(E)** and CRC tumor tissues **(F)**. **(G)** Pseudotime trajectory analysis of T cells, depicting differentiation into 11 cellular states along pseudotime. **(H)** Heatmap showing dynamic expression patterns of prognostic genes (SIX1, NOX4, CDKN2A, and CXCL1) along the T-cell pseudotime trajectory. **(I)** Dot plot illustrating activity levels of multiple metabolic pathways across different cell types. **(J)** Integrated pathway enrichment analysis (irGSEA) across multiple algorithms for different cell types.

Cell–cell communication analysis revealed that T cells exhibited extensive interactions with multiple cell types (**Figures 7A, B**). In normal tissues, the most prominent interactions involved B cells and macrophage/monocyte populations (**Figure 7C**). In CRC tumor tissues, T cells showed frequent interactions with plasmacytoid dendritic cells, endothelial cells, and macrophages/monocytes (**Figure 7D**). Among these interactions, the ligand-receptor pair MIF-(CD74 + CXCR4) displayed the highest communication probability in both tumor and normal tissues (**Figures 7E, F**), highlighting a potential T-cell-centered communication axis in the CRC microenvironment. Further clustering analysis of T cells identified seven subclusters based on the top 30 principal components (**Supplementary Figure S10A–C**). Pseudotime trajectory analysis divided T cells into 11 distinct states along the differentiation trajectory (**Figure 7G**). Cluster 1 and cluster 3 were predominantly distributed at early pseudotime stages, cluster 0 was mainly located at intermediate stages, and cluster 2 was enriched at later stages. Dynamic expression analysis along pseudotime demonstrated that SIX1, NOX4, and CXCL1 exhibited higher expression levels at early stages, and then gradually declined, whereas CDKN2A expression increased progressively toward later states (**Figure 7H**). Single-cell metabolic and functional enrichment analyses revealed differences in pathway activity among cell types (**Figure 7I**). In T cells, several metabolic pathways, including propanoate metabolism and fatty acid elongation, showed relatively higher activity. Integrated pathway enrichment analysis across multiple algorithms further showed that T cells were enriched for immune-related and cell cycle-related gene sets, including allograft rejection, MYC target gene signatures, and the G2M checkpoint, whereas pathways related to epithelial-mesenchymal transition and myogenesis showed lower activity(**Figure 7J**). Together, these findings place T cells in a communication-active, metabolically remodeled, and functionally dynamic context within CRC.

### 3.9 NOX4-centered functional validation supports a redox/inflammatory component of the SCore-associated state

**Figure 8:**
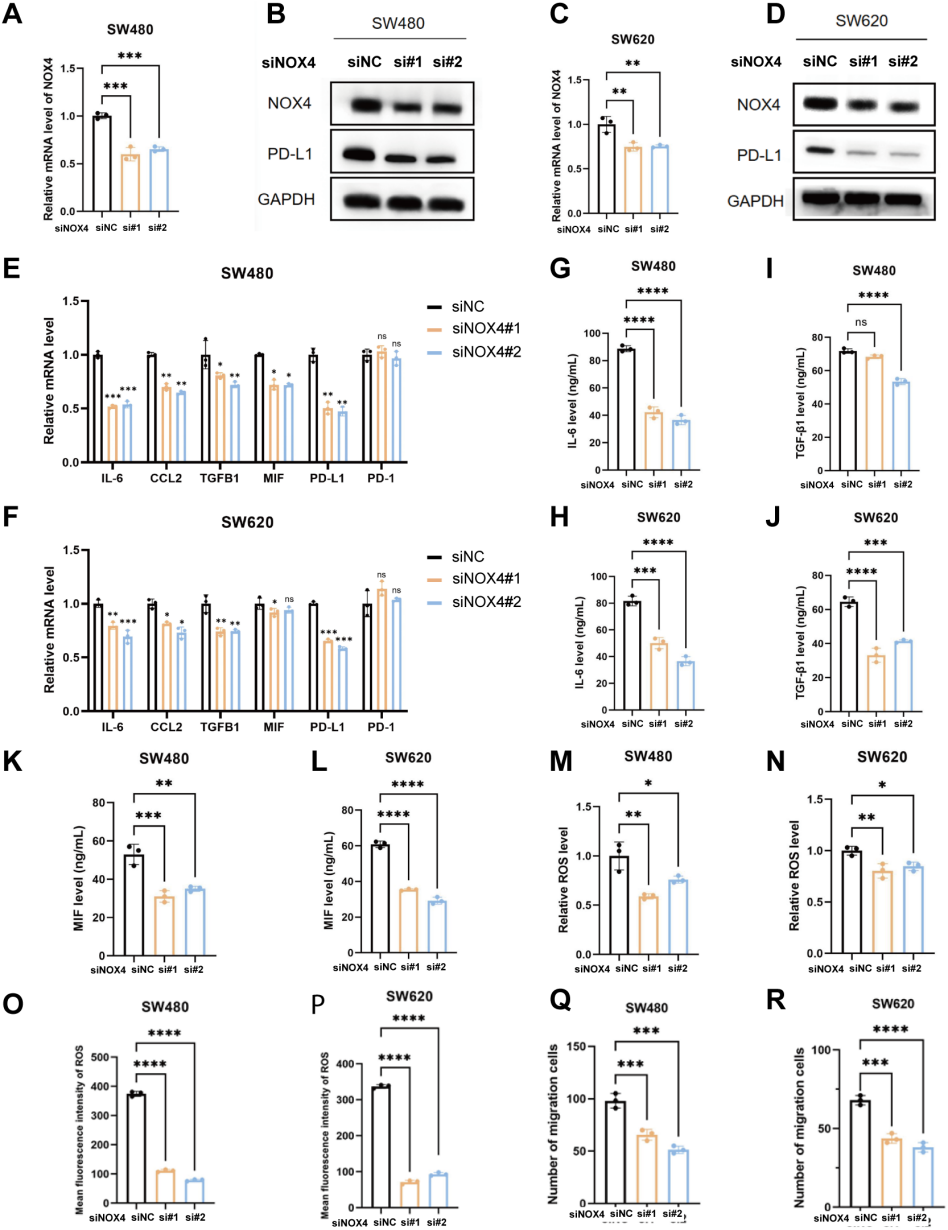
NOX4 knockdown attenuates inflammatory and immunoregulatory outputs, reduces intracellular ROS levels, and impairs migratory capacity in CRC cells. **(A–D)** Validation of NOX4 knockdown efficiency in SW480 and SW620 cells. **(A, C)** Relative NOX4 mRNA expression measured by qPCR. **(B, D)** Western blot analysis of NOX4 and PD-L1 protein levels. **(E, F)** Transcriptional changes in inflammatory and immunoregulatory genes following NOX4 knockdown. **(E)** SW480 cells; **(F)** SW620 cells. Genes analyzed include IL-6, CCL2, TGFB1, MIF, PD-L1, and PD-1. **(G–L)** Cytokine secretion in culture supernatants measured by ELISA after NOX4 knockdown. **(G, H)** IL-6; **(I, J)** TGF-β1; **(K, L)** MIF. **(M, N)** Relative intracellular reactive oxygen species (ROS) levels in SW480 and SW620 cells following NOX4 knockdown. **(O, P)** Quantification of ROS fluorescence intensity. **(O)** SW480 cells; **(P)** SW620 cells. **(Q, R)** Transwell migration assays showing reduced migratory capacity upon NOX4 knockdown. **(Q)** SW480 cells; **(R)** SW620 cells. ns, not significant; *P < 0.05; **P < 0.01; ***P < 0.001; ****P < 0.0001.

To provide functional support beyond baseline expression analyses, we performed the main validation experiments in SW480 and SW620 cells. qPCR and western blotting confirmed efficient NOX4 knockdown at both the mRNA and protein levels, and PD-L1 protein expression was concurrently reduced after NOX4 silencing (**Figures 8A–D**). We next examined a panel of inflammatory and immunoregulatory mediators. qPCR showed that NOX4 knockdown significantly decreased the expression of IL-6, CCL2, TGFB1, MIF, and PD-L1, whereas PD-1 showed no obvious change (**Figures 8E, F**). ELISA further demonstrated reduced secretion of IL-6, TGF-β1 and MIF in culture supernatants after NOX4 knockdown (**Figures 8G–L**). Given the established role of NOX4 in redox regulation, we next assessed intracellular reactive oxygen species (ROS). NOX4 silencing significantly reduced intracellular ROS levels in both SW480 and SW620 cells, as confirmed by fluorescence imaging (**Figures 8M–P**; **Supplementary Figure 11A**). In addition, Transwell migration assays demonstrated that NOX4 knockdown significantly reduced the number of migrated tumor cells, indicating that NOX4 supports the migratory phenotype of CRC cells (**Figures 8Q, R**; **Supplementary Figure 11B**). To determine whether this NOX4-associated redox/inflammatory phenotype could also be observed in additional CRC models, we further performed transient NOX4 knockdown in HCT15 and HCT116 cells. In these cells, NOX4 silencing similarly reduced intracellular ROS and altered the expression of inflammatory/immunoregulatory mediators, including IL-6, CCL2, TGFB1, and PD-L1 (**Supplementary Figures 11C–F**). Collectively, these findings provide functional support for a NOX4-centered redox/inflammatory component within the broader SCore-associated state.

## 4 Discussion

Colorectal cancer (CRC) remains biologically and clinically heterogeneous, and this heterogeneity is not fully captured by conventional clinicopathological staging. Both senescence-associated programs and circadian disruption have been implicated in adverse CRC biology, including invasion, metastasis, immune dysfunction, therapeutic resistance, and poor clinical outcome (21, 22, 66). These processes are increasingly recognized as mechanistically interconnected rather than independent (20, 67). On this basis, we focused on the transcriptional overlap between senescence- and circadian-related programs and developed an externally validated random survival forest-derived senescence–circadian interplay score (SCore). SCore stratified survival and was associated with oxidative stress, extracellular matrix remodeling, and computationally inferred immune dysfunction/exclusion, with additional support from single-cell analyses and NOX4-centered functional experiments. Collectively, these findings define a stress-regulatory axis linking senescence–circadian overlap to CRC progression and tumor–microenvironment interactions.

SCore should not be interpreted as a direct quantitative measure of mechanistic senescence–circadian coupling. Instead, it serves as a transcriptomic proxy for the overlap between these programs. The high-SCore state was characterized by coordinated enrichment of oxidative stress, focal adhesion, ECM–receptor interaction, and invasion-related pathways, whereas canonical circadian pathways were not dominant. This pattern indicates that the identified overlap is expressed as a broader stress-adaptive malignant state rather than a discrete clock signature. This view is consistent with recent evidence showing that physiological ageing promotes metastasis through activation of the integrated stress response and ATF4-driven epithelial and metabolic plasticity (68). Although demonstrated in lung adenocarcinoma, this supports a general model in which stress-adaptive programs enhance tumor progression through plasticity-driven reprogramming. Enrichment of aldosterone-regulated sodium reabsorption further supports activation of a mineralocorticoid/ion-transport network (NR3C2, SGK1, ENaC, NEDD4L) that may contribute to stress adaptation (69). Overall, the high-SCore state reflects coordinated redox imbalance, tumor–matrix interaction, and invasive niche remodeling (14, 70).

The derivation of SCore is consistent with this interpretation. From 278 senescence-related genes and 2,095 circadian-related genes, 75 overlapping ACR genes were identified. Intersection with CRC-associated differentially expressed genes yielded 10 candidates, from which four prognostic genes (CXCL1, CDKN2A, SIX1, and NOX4) were retained through Cox and RSF modeling. Although these genes are not canonical clock components or classical senescence markers, they converge on stress signaling, redox imbalance, inflammatory output, and tumor progression. Consistent with these findings, tissue-microarray immunohistochemistry further confirmed increased protein expression of all four genes in CRC compared with paired adjacent tissues, providing tissue-level support complementary to transcriptomic and *in vitro* data.

Among the four genes, NOX4 emerged as the most compelling functional anchor. NOX4-derived ROS has been implicated in senescence maintenance, inflammatory signaling, immune regulation, and treatment resistance (71, 72), and circadian disruption may further amplify these stress programs through dysregulated redox control (73). In our experiments, NOX4 knockdown in SW480 and SW620 cells reduced intracellular ROS, suppressed both the expression and secretion of IL-6, CCL2, TGF-β1, MIF, and PD-L1, and impaired migratory capacity. These findings are broadly consistent with prior evidence linking NOX4-derived ROS to pro-inflammatory signaling and immune-regulatory outputs, while ROS itself also contributes to senescence-associated stress signaling (74–76). The reduction in CCL2 and MIF further suggests that NOX4 may modulate the ability of tumor cells to recruit and condition immune cells, thereby contributing to immunosuppressive microenvironmental features (77, 78). Although these data remain limited to *in vitro* knockdown experiments, they place NOX4 lies at an intersection of redox stress, inflammatory signaling, and circadian-associated tumor–immune dysregulation.

The remaining three genes are likewise compatible with the biological state represented by high SCore, although their roles appear to be more context-dependent. CDKN2A is a core regulator of cellular senescence and can promote chronic inflammation and stromal remodeling through the SASP when senescent cells persist (79–81). SIX1 is closely associated with EMT, angiogenesis, metastasis, and stromal remodeling, and may cooperate with inflammatory and matrix-remodeling programs to drive aggressive tumor behavior (82–84). We also observed marked expression heterogeneity of CDKN2A and SIX1 across CRC cell lines: CDKN2A was downregulated in DLD1 but upregulated in HCT15, HT29, and RKO, whereas SIX1 was downregulated in most lines. This variability may reflect differences in molecular subtype and driver mutation background, as DLD1 and HCT15 are MSI-H with PIK3CA mutations, whereas SW480, HT29, and RKO are predominantly MSS with distinct TP53 or KRAS/BRAF alterations (85–87). These observations indicate that CDKN2A- and SIX1-related effects should be interpreted in the context of cell-line molecular background during functional validation. By contrast, CXCL1 appears more complex. It is well established that CXCL1 participates in myeloid recruitment and senescence-associated inflammation through the CXCL1-CXCR2 axis (88–90), yet in our univariate Cox analysis it showed a protective trend. This apparent paradox may reflect the coexistence of protumor signaling and incomplete immune compensation within the same tumor ecosystem. This apparent paradox likely reflects coexistence of protumor signaling and incomplete immune compensation within the same tumor ecosystem.

The immune analyses indicate that SCore captures functional immune remodeling rather than simple differences in immune-cell abundance. Correlations of CXCL1 with neutrophils, NOX4 with activated dendritic cells, and CDKN2A with CD56^dim NK cells suggest coordinated shifts in immune recruitment and effector-state composition across risk strata. High-SCore tumors also showed higher TIDE scores and altered expression of multiple immune checkpoint-related genes, consistent with the presence of a more immune-dysfunctional or immune-excluded niche. This interpretation is particularly relevant in MSS CRC, where checkpoint blockade is often limited by a suppressive immune context rather than by a single dominant resistance pathway (91–93).

The relationship between SCore and CMS classification was also notable. SCore was significantly higher in CMS1 and CMS4 than in CMS2 and CMS3. This pattern is consistent with recognized heterogeneity within CMS1. CMS1 is commonly regarded as an immune-infiltrated subtype whereas CMS4 is associated with mesenchymal biology and poor prognosis (94). A subset of CMS1 tumors exhibits CMS4-like mesenchymal features accompanied by chronic inflammation, oxidative stress, and increased expression of inflammatory mediators and immune checkpoint molecules (95). These findings indicate that SCore may refine CMS-based stratification by identifying tumors in which immune-related and stromal/stress-associated adverse features coexist.

The single-cell analyses provide an important cellular perspective on these findings. SCore-associated programs localized to a T-cell-centered context, not simply due to abundance but because risk-associated gene expression, cell–cell communication, and pseudotime dynamics converged in this population. Along the trajectory, SIX1, NOX4, and CXCL1 were higher in early states, whereas CDKN2A increased later, consistent with a transition toward more senescence-like or dysfunctional states. High-SCore T cells were enriched for exhaustion markers (PDCD1, LAG3, HAVCR2) together with CDKN2A, supporting a link between senescence-associated stress and T-cell dysfunction (96, 97). The dynamic behavior of SCore along pseudotime further supports a continuous remodeling process rather than a fixed state.

The communication and metabolic features of the T-cell compartment further reinforce this interpretation. The prominence of MIF-(CD74+CXCR4) is of particular interest because this axis has been implicated in immune-cell migration, activation, and suppressive immune ecology, including effects on activated T cells and tumor-infiltrating regulatory T cells (98, 99). Similarly, the relatively higher activity of fatty acid elongation is biologically meaningful, given increasing evidence that lipid metabolic dysregulation shapes T-cell activation, differentiation, dysfunction, and resistance to immunotherapy in tumors (100–102). Taken together, these observations support that T cells in high-SCore tumors are associated with coordinated immunometabolic remodeling, altered intercellular communication, and progressive functional-state transition. This interpretation is also consistent with our NOX4-centered functional data, in which perturbation of a redox/inflammatory node reduced ROS and multiple immunoregulatory outputs, thereby supporting a stress-linked mechanism that may contribute to the immune context captured by SCore.

The drug-sensitivity analysis provides an additional translational dimension. Among the 73 agents with significantly different predicted IC_50_ values, several drugs directly relevant to CRC treatment—including 5-fluorouracil, oxaliplatin, irinotecan, paclitaxel, docetaxel, mitomycin, and teniposide—showed higher predicted IC_50_ values in the high-SCore group, suggesting reduced sensitivity or a greater propensity toward treatment resistance. This pattern is broadly consistent with prior work linking circadian dysregulation and senescence-associated programs to altered chemotherapy response. For example, ARNTL2 has been shown to promote resistance to 5-fluorouracil by upregulating SLC7A11 and suppressing ferroptosis (103), whereas senescence escape after irinotecan exposure may favor expansion of more invasive and chemoresistant clones (104). In oxaliplatin-resistant settings, including CRC, resistance has been associated with enhanced oxidative phosphorylation, immune-regulatory changes, and stromal remodeling (105–107). These observations are broadly concordant with the stress, matrix, and immune features associated with the high-SCore state, although the translational relevance of this association will require direct clinical validation.

From a translational perspective, SCore may provide a useful framework for molecular stratification in colorectal cancer. High-SCore tumors exhibited concurrent features of immune dysfunction/exclusion, extracellular matrix remodeling, and a trend toward reduced predicted drug sensitivity, suggesting a composite state marked by tumor-intrinsic stress, stromal remodeling, and changes in immune context. These findings indicate that high-SCore tumors may be less responsive to immunotherapy or standard chemotherapy, whereas combination strategies targeting senescence-associated secretory programs or stromal remodeling merit further investigation, pending prospective validation. Compared with CMS classification or single-gene signatures, SCore integrates nonlinear interactions among senescence- and circadian-related genes and may therefore capture immune-stromal co-dysregulation not fully resolved by conventional subtyping alone. In addition, the nomogram incorporating SCore, age, and M stage provides a continuous estimate of survival probability and may therefore be more informative than simple risk dichotomization for individualized follow-up and adjuvant treatment planning. At present, however, SCore is best regarded as a hypothesis-generating framework for risk stratification and study design rather than a tool ready for routine clinical use.

Several limitations should be acknowledged. First, the analyses were based primarily on retrospective public cohorts and may therefore be subject to unmeasured confounding and cohort-specific biases. Second, treatment-response annotations were limited and, because suitable public datasets were scarce, we were unable to directly evaluate the predictive value of SCore for immune checkpoint inhibitor efficacy in a large independent colorectal cancer cohort treated with immunotherapy. Accordingly, whether SCore can serve as a biomarker of immunotherapy benefit remains to be established in prospective clinical studies. Third, although the NOX4 knockdown experiments provide orthogonal support for a tumor-cell-intrinsic redox and inflammatory component, they do not establish a complete causal chain linking circadian-associated dysregulation, senescence-related stress, and downstream T-cell dysfunction. Fourth, the predictive value of the model for immunotherapy, chronotherapy, and senescence-targeting strategies requires prospective validation. Finally, whether the association of SCore with adverse prognosis and immune remodeling is causally contributory or primarily reflective of broader underlying tumor states remains unresolved and will require future functional studies, including co-culture systems and CRISPR-based perturbation experiments.

In summary, we developed and externally validated an RSF-derived scoring system, SCore, that captures a senescence-circadian interplay-associated transcriptional state in CRC. Beyond prognostic stratification, SCore tracks coordinated remodeling of the tumor microenvironment, with T cells emerging as a key cellular context. These findings support a model in which senescence-associated stress and circadian dysregulation converge on redox, stromal, and immune remodeling programs, contributing to CRC aggressiveness and survival heterogeneity.

## Data Availability

The datasets analyzed in this study are publicly available from TCGA and GEO. The TCGA colorectal cancer cohorts, including TCGA-COAD and TCGA-READ, are available via the NCI Genomic Data Commons. The GEO datasets analyzed in this study include GSE12945, GSE39582, and GSE132465. Analysis code is deposited in GitHub at https://github.com/luolz/CRC-senescence-circadian-interplay. All other data supporting the findings of this study are available from the corresponding authors upon reasonable request.

## Supplementary material

Supplementary Figure 1 Research flowchart.

Supplementary Figure 2 Proportional hazards assumption diagnostics and SCore distribution in training and external validation cohorts. **(A)** Schoenfeld residual plot for CDKN2A evaluating the proportional hazards (PH) assumption in the univariate Cox regression analysis. **(B)** Schoenfeld residual plot for CXCL1 assessing the PH assumption. **(C)** Schoenfeld residual plot for NOX4 showing the relationship between scaled Schoenfeld residuals and time. **(D)** Schoenfeld residual plot for SIX1 used to assess the PH assumption in the Cox regression model. **(E)** Distribution of SCore and survival status of CRC patients in the training cohort. The upper panel shows SCore ranked in ascending order, with the dashed line indicating the optimal cutoff value. The lower panel displays corresponding survival status and survival time. **(F)** Distribution of SCore and survival status of CRC patients in the external validation cohort GSE12945.

Supplementary Figure 3 Validation of the risk model. **(A)** K-M survival curve **(B)** Risk curve **(C)** ROC curve of the validation set.

Supplementary Figure 4 Validation of prognostic gene expression and its association with oxidative stress and inflammatory pathways. **(A, B)** Relative mRNA expression levels of these four prognostic genes in normal colon epithelial cell line NCM460 and colorectal cancer cell lines DLD1, SW480, HCT15, HT29, and RKO, determined by reverse transcription quantitative PCR (RT-qPCR): CDKN2A **(A)**, SIX1 **(B)**. **(C, D)** Quantitative immunohistochemical validation of CDKN2A and SIX1 expression in the SYSUCC-CRC tissue microarray. IHC scores were significantly higher in colorectal cancer tissues than in paired adjacent non-tumor tissues (n = 120). **(E)** Representative immunohistochemical staining images of NOX4, CDKN2A, CXCL1, and SIX1 in paired colorectal cancer tissues (T) and adjacent non-tumor tissues (ANT). Scale bar, 50 µm. The corresponding case IDs for the representative images are indicated in **Supplementary Table 6**. **(F)** Bar plot of the number of significantly co-expressed genes between prognostic genes and pathway genes. The bar plot shows the number of significantly co-expressed genes between each of the four prognostic genes and the oxidative stress pathway or the SASP inflammatory response pathway. Co-expression significance threshold: Spearman correlation coefficient |rho| ≥ 0.3 and BH-adjusted FDR < 0.05.

Supplementary Figure 5 Associations between clinicopathological characteristics and prognostic SCore in CRC. **(A)** Comparison of SCore between patients with M0 and M1 disease. **(B)** Distribution of SCore across T stages (T1-T4). **(C)** Comparison of SCore among pathological stages (stage I, stage II, and stage III/IV). **(D)** Comparison of SCore between age groups (< 60 years and ≥ 60 years). **(E)** Comparison of SCore between female and male patients. Statistical significance: *P < 0.05; **P < 0.01; ***P < 0.001; ns, not significant.

Supplementary Figure 6 Quality control of single-cell RNA sequencing data from the GSE132465 dataset. **(A)** Violin plots showing the distributions of quality control metrics, including the number of detected genes per cell (nFeature_RNA), total RNA counts per cell (nCount_RNA), and the percentage of mitochondrial gene expression (percent.mt), before quality control filtering. **(B)** Violin plots showing the distributions of nFeature_RNA, nCount_RNA, and percent.mt after quality control filtering. E) Expression of core genes in T−cell subpopulations. Dot plot showing expression levels of CXCL1, CDKN2A, NOX4, and SIX1 in T−cell subpopulations. Bluer color indicates higher expression level, and larger circle size indicates a higher proportion of cells expressing the gene.

Supplementary Figure 7 Annotation of T-cell subpopulations and validation of expression levels. **(A)** PCA results of the single-cell dataset. Left panel: PCA result showing the dimensionality reduction process using principal component analysis; each line represents one principal component. Right panel: Scree plot, with the x−axis indicating principal components and the y-axis indicating standard deviation, which describes the dispersion or variability of the data. **(B)** Clustering analysis results. **(C)** Cell annotation results. **(D)** Expression of marker genes for T-cell subpopulation annotation. The x-axis shows marker genes, and the y-axis shows cell types. Larger and bluer circles indicate higher gene expression in the cells. **(E)** Expression of core genes in T−cell subpopulations. Dot plot showing expression levels of CXCL1, CDKN2A, NOX4, and SIX1 in T-cell subpopulations. Bluer color indicates higher expression level, and larger circle size indicates a higher proportion of cells expressing the gene.

Supplementary Figure 8 SCore-associated T-cell expression states. **(A)** Differential expression of exhaustion/senescence markers in T cells between high- and low- SCore groups. Expression distributions in high-SCore versus low−SCore T cells. Blue represents the low−SCore group, and red represents the high−SCore group. **(B)** Dot plot of marker expression stratified by high/low SCore across different T-cell subpopulations. Color indicates average expression, and dot size represents the proportion of cells expressing the marker. **(C)** Distribution of T-cell SCore in the reduced dimensional space. Continuous distribution of T-cell SCore shown in t−SNE space, reflecting the spatial aggregation of cells with high functional scores within the population.

Supplementary Figure 9 SCore-associated pseudotime analysis of T cells. **(A)** Pseudotime trajectory of T cells and SCore mapping. Top left: Colored by pseudotime, showing the direction of differentiation progression. Top right: Colored by continuous SCore, showing the spatial distribution of SCore along the trajectory. Bottom left: Colored by SCore_group (High/Low), showing enrichment differences of high and low groups in different regions of the trajectory. Bottom right: Colored by T_subtype, displaying the distribution pattern of subpopulations along the differentiation path and potential branch preferences. **(B)** Dynamic changes along T- cell pseudotime. **(C)** Comparison of pseudotime trajectories between high- and low-SCore T−cell groups. **(D)** Co-expression dynamics of key markers along pseudotime.

Supplementary Figure 10 Dimensionality reduction and subclustering analysis of T cells in the single-cell RNA sequencing dataset. **(A)** Principal component analysis (PCA) significance assessment of T cells using JackStraw analysis. **(B)** PCA scree plot showing the variance explained by each principal component in T cells. **(C)** t-distributed stochastic neighbor embedding (t-SNE) plot illustrating subclustering of T cells based on selected principal components.

Supplementary Figure 11 Representative imaging and additional validation of NOX4 knockdown effects. **(A)** Representative fluorescence images showing intracellular ROS levels in SW480 and SW620 cells following NOX4 knockdown. **(B)** Representative images of Transwell migration assays in SW480 and SW620 cells after NOX4 knockdown. **(C, D)** qPCR analysis of IL-6, CCL2, TGF-β1, and PD-L1 mRNA levels following transient NOX4 knockdown in HCT15 and HCT116 cells. **(E, F)** Quantification of intracellular ROS levels in HCT15 and HCT116 cells after NOX4 knockdown.

Supplementary Table 1 List of 75 genes associated with both cellular senescence and circadian rhythm.

Supplementary Table 2 Reverse transcription-quantitative PCR (RT-qPCR) cycling conditions used for expression analysis of prognostic genes.

Supplementary Table 3 Primer sequences for the prognostic genes used in RT-qPCR analysis.

Supplementary Table 4 Gene ontology and Kyoto encyclopedia of genes and genomes (KEGG) pathway enrichment analyses of the 10 senescence- and circadian rhythm-related candidate genes.

Supplementary Table 5 Clinicopathological characteristics of the SYSUCC-CRC tissue microarray cohort.

Supplementary Table 6 Immunohistochemical scores of NOX4, CXCL1, CDKN2A, and SIX1 in paired colorectal cancer and adjacent non-tumor tissues.

Supplementary Table 7 Gene set enrichment analysis results for pathways associated with prognostic SCore groups and individual prognostic genes in CRC.

## Glossary

ACR: Aging-Circadian Rhythm intersection
AUC: Area under the curve
BP: Biological process
CC: Cellular component
CHF: Cumulative hazard function
CMS: Consensus molecular subtype
COAD: Colon adenocarcinoma
CRC: Colorectal cancer
DEGs: Differentially expressed genes
FC: Fold change
GDSC: Genomics of Drug Sensitivity in Cancer
GEO: Gene Expression Omnibus
GO: Gene Ontology
GSEA: Gene set enrichment analysis
GSVA: Gene set variation analysis
HR: Hazard ratio
SCore: Senescence-Circadian Interplay Score
HSG: High-SCore group
IC_50_: Half maximal inhibitory concentration
KEGG: Kyoto Encyclopedia of Genes and Genomes
LSG: Low-SCore group
MF: Molecular function
NES: Normalized enrichment score
PCA: Principal component analysis
pDCs: Plasmacytoid dendritic cells
PH: Proportional hazards
PPI: Protein-protein interaction
READ: Rectum adenocarcinoma
ROC: Receiver operating characteristic
RRA: Robust rank aggregation
RSF: Random survival forest
SASP: Senescence-associated secretory phenotype
scRNA-seq: Single-cell RNA sequencing
ssGSEA: Single-sample gene set enrichment analysis
ANT: Adjacent non-tumor tissue
TCGA: The Cancer Genome Atlas
TIDE: Tumor Immune Dysfunction and Exclusion
TMB: Tumor mutational burden
TME: Tumor microenvironment
IHC: Immunohistochemistry
t-SNE: t-distributed stochastic neighbor embedding
RT-qPCR: Reverse transcription quantitative PCR
ATCC: American Type Culture Collection
