# Supplementary figures and images for: Senescence-circadian interplay stratifies patient prognosis and reveals immune remodeling heterogeneity in colorectal cancer
Source: Front Immunol. 2026 Jun 3;17:1804974. doi: 10.3389/fimmu.2026.1804974 (PMC13272428; doi:10.3389/fimmu.2026.1804974)

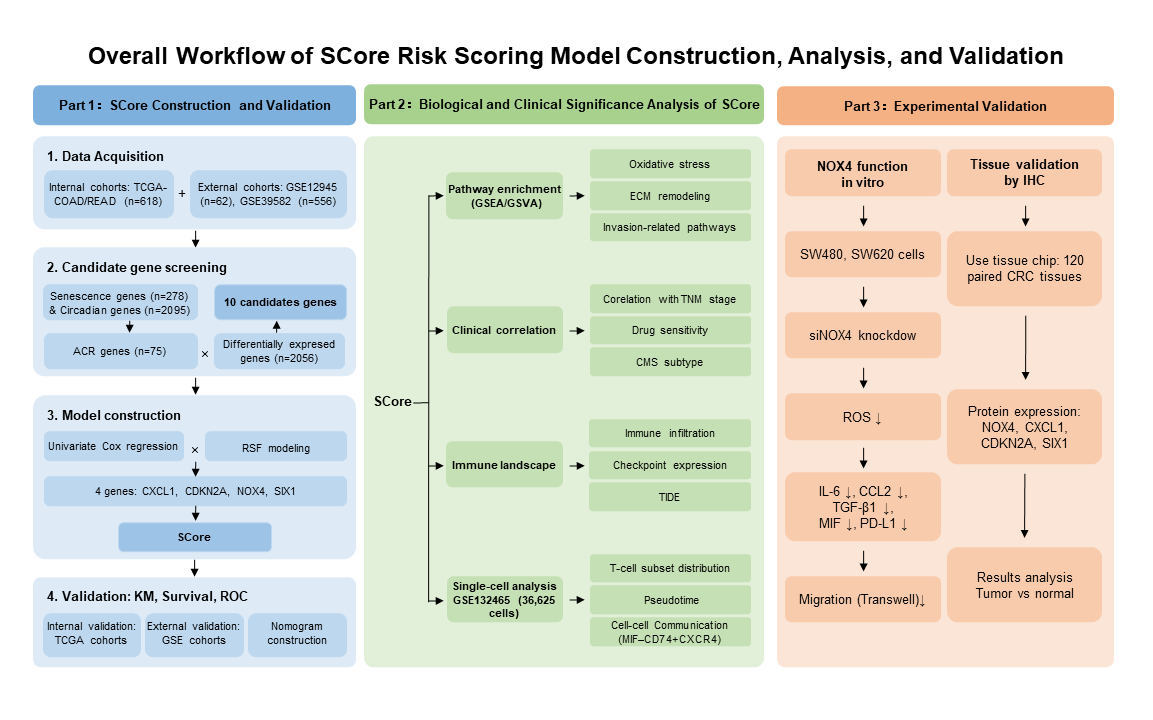

Supplement: Supplementary Figure 1 — Research flowchart. [file Image1.tif]

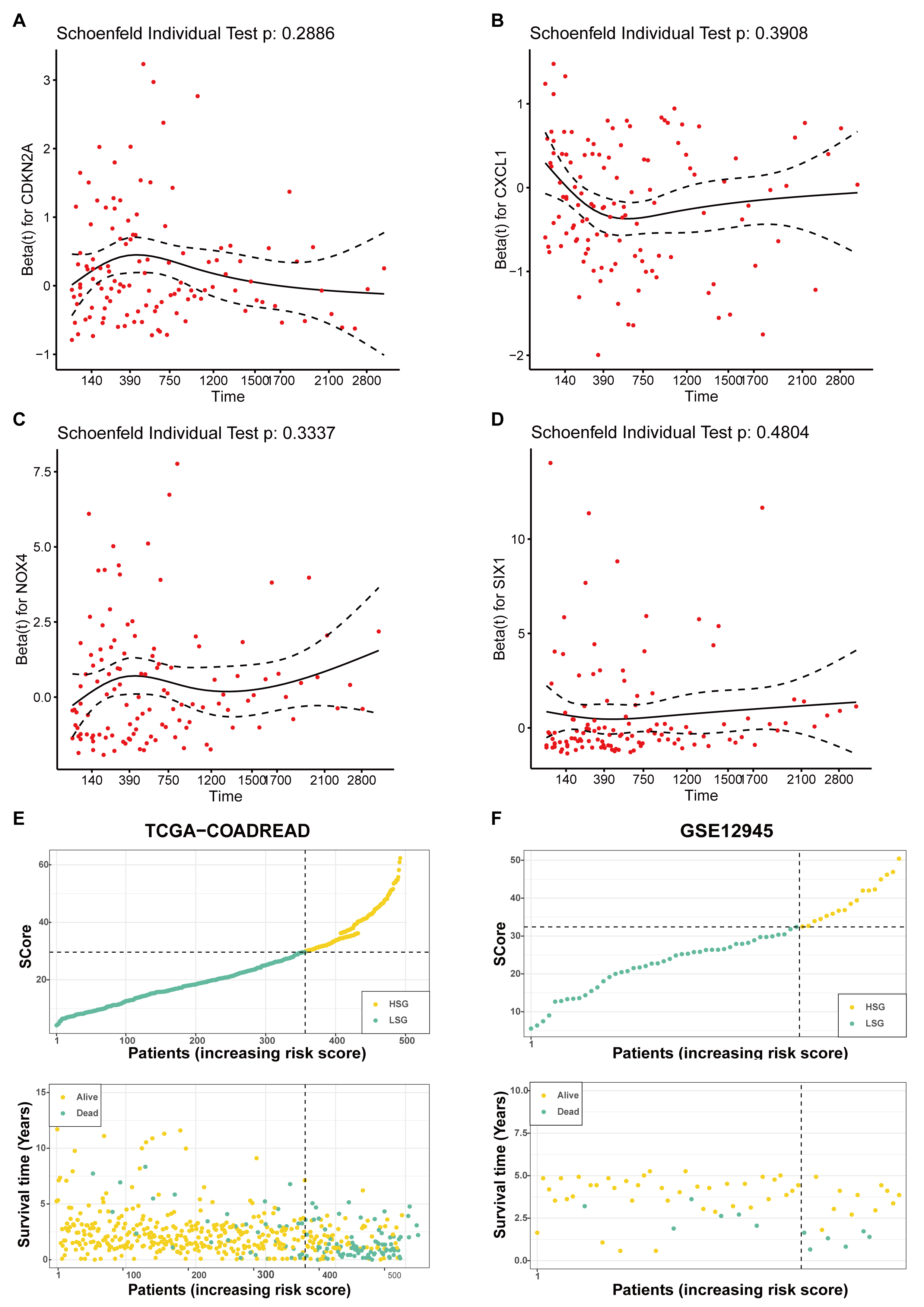

Supplement: Supplementary Figure 2 — Proportional hazards assumption diagnostics and SCore distribution in training and external validation cohorts. (A) Schoenfeld residual plot for CDKN2A evaluating the proportional hazards (PH) assumption in the univariate Cox regression analysis. (B) Schoenfeld residual plot for CXCL1 assessing the PH assumption. (C) Schoenfeld residual plot for NOX4 showing the relationship between scaled Schoenfeld residuals and time. (D) Schoenfeld residual plot for SIX1 used to assess the PH assumption in the Cox regression model. (E) Distribution of SCore and survival status of CRC patients in the training cohort. The upper panel shows SCore ranked in ascending order, with the dashed line indicating the optimal cutoff value. The lower panel displays corresponding survival status and survival time. (F) Distribution of SCore and survival status of CRC patients in the external validation cohort GSE12945. [file Image2.tif]

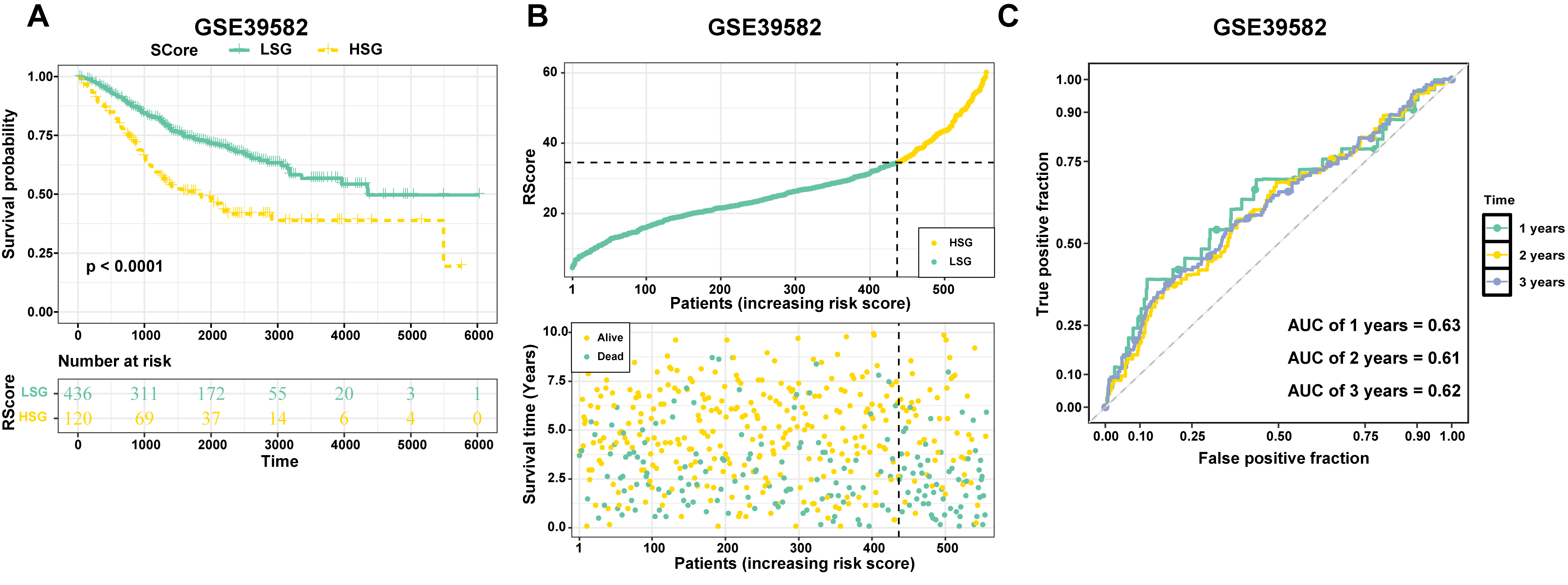

Supplement: Supplementary Figure 3 — Validation of the risk model. (A) K-M survival curve (B) Risk curve (C) ROC curve of the validation set. [file Image3.tif]

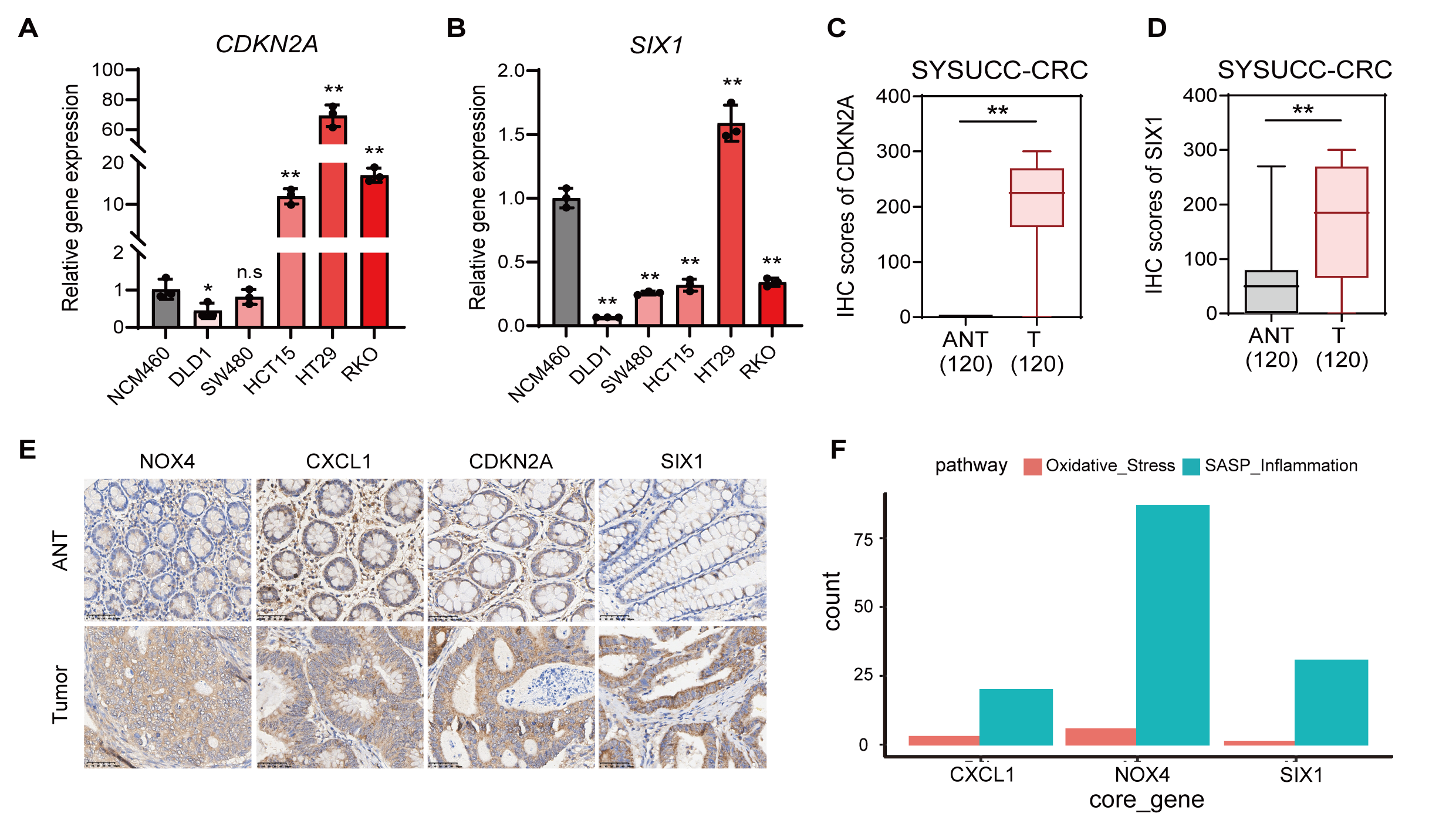

Supplement: Supplementary Figure 4 — Validation of prognostic gene expression and its association with oxidative stress and inflammatory pathways. (A, B) Relative mRNA expression levels of these four prognostic genes in normal colon epithelial cell line NCM460 and colorectal cancer cell lines DLD1, SW480, HCT15, HT29, and RKO, determined by reverse transcription quantitative PCR (RT-qPCR): CDKN2A (A), SIX1 (B). (C, D) Quantitative immunohistochemical validation of CDKN2A and SIX1 expression in the SYSUCC-CRC tissue microarray. IHC scores were significantly higher in colorectal cancer tissues than in paired adjacent non-tumor tissues (n = 120). (E) Representative immunohistochemical staining images of NOX4, CDKN2A, CXCL1, and SIX1 in paired colorectal cancer tissues (T) and adjacent non-tumor tissues (ANT). Scale bar, 50 µm. The corresponding case IDs for the representative images are indicated in Supplementary Table 6. (F) Bar plot of the number of significantly co-expressed genes between prognostic genes and pathway genes. The bar plot shows the number of significantly co-expressed genes between each of the four prognostic genes and the oxidative stress pathway or the SASP inflammatory response pathway. Co-expression significance threshold: Spearman correlation coefficient |rho| ≥ 0.3 and BH-adjusted FDR < 0.05. [file Image4.tif]

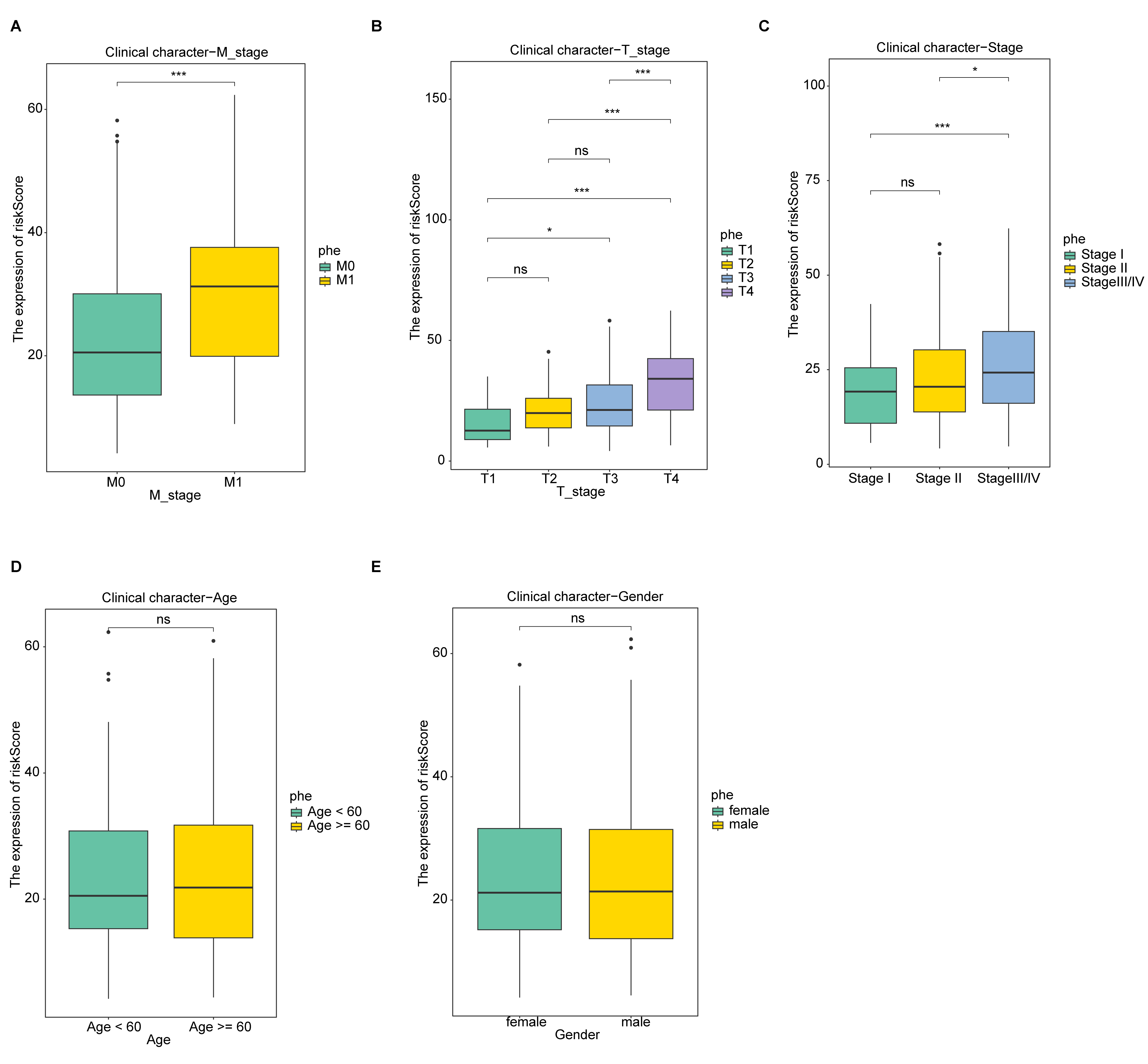

Supplement: Supplementary Figure 5 — Associations between clinicopathological characteristics and prognostic SCore in CRC. (A) Comparison of SCore between patients with M0 and M1 disease. (B) Distribution of SCore across T stages (T1-T4). (C) Comparison of SCore among pathological stages (stage I, stage II, and stage III/IV). (D) Comparison of SCore between age groups (< 60 years and ≥ 60 years). (E) Comparison of SCore between female and male patients. Statistical significance: *P < 0.05; **P < 0.01; ***P < 0.001; ns, not significant. [file Image5.tif]

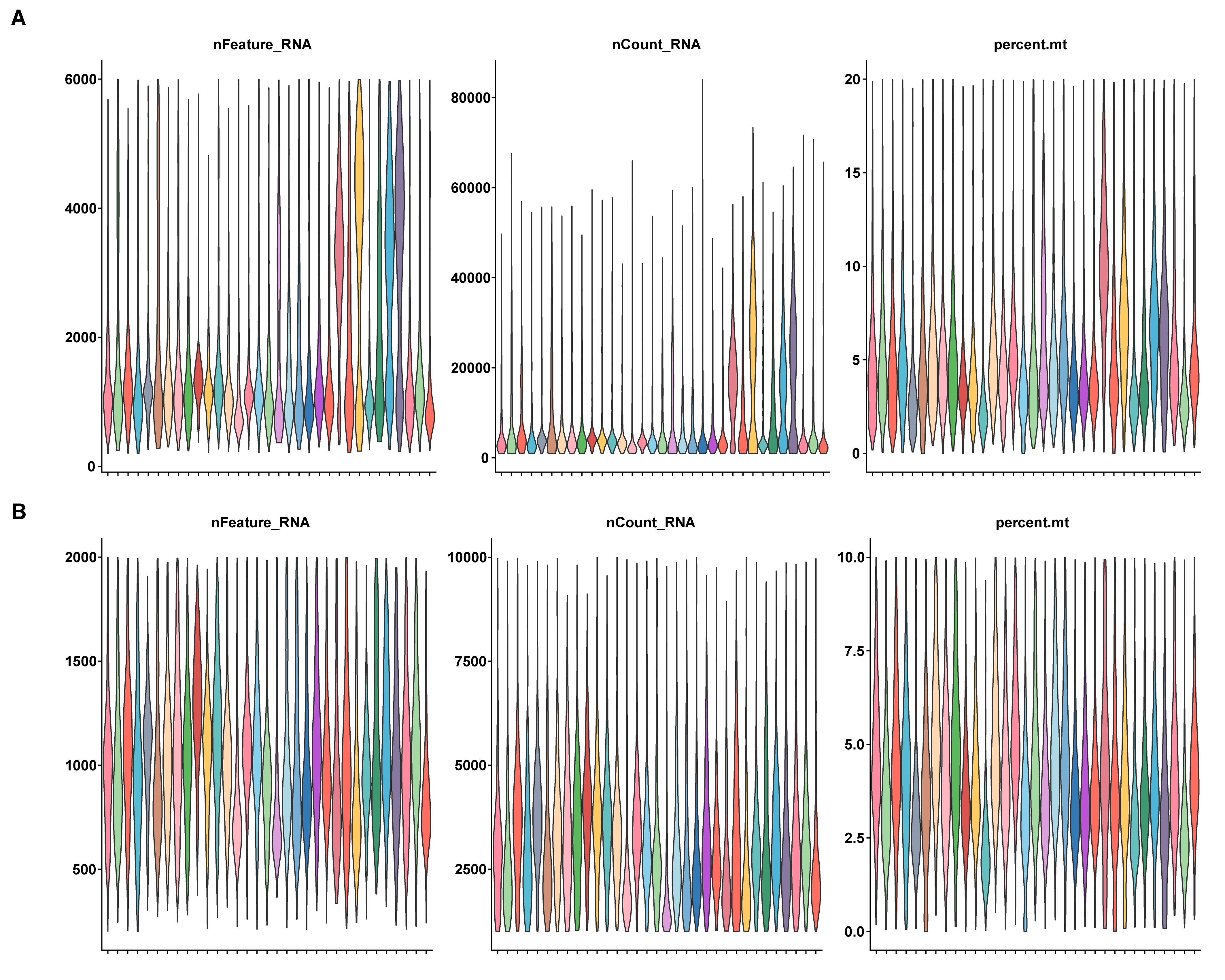

Supplement: Supplementary Figure 6 — Quality control of single-cell RNA sequencing data from the GSE132465 dataset. (A) Violin plots showing the distributions of quality control metrics, including the number of detected genes per cell (nFeature_RNA), total RNA counts per cell (nCount_RNA), and the percentage of mitochondrial gene expression (percent.mt), before quality control filtering. (B) Violin plots showing the distributions of nFeature_RNA, nCount_RNA, and percent.mt after quality control filtering. E) Expression of core genes in T−cell subpopulations. Dot plot showing expression levels of CXCL1, CDKN2A, NOX4, and SIX1 in T−cell subpopulations. Bluer color indicates higher expression level, and larger circle size indicates a higher proportion of cells expressing the gene. [file Image6.tif]

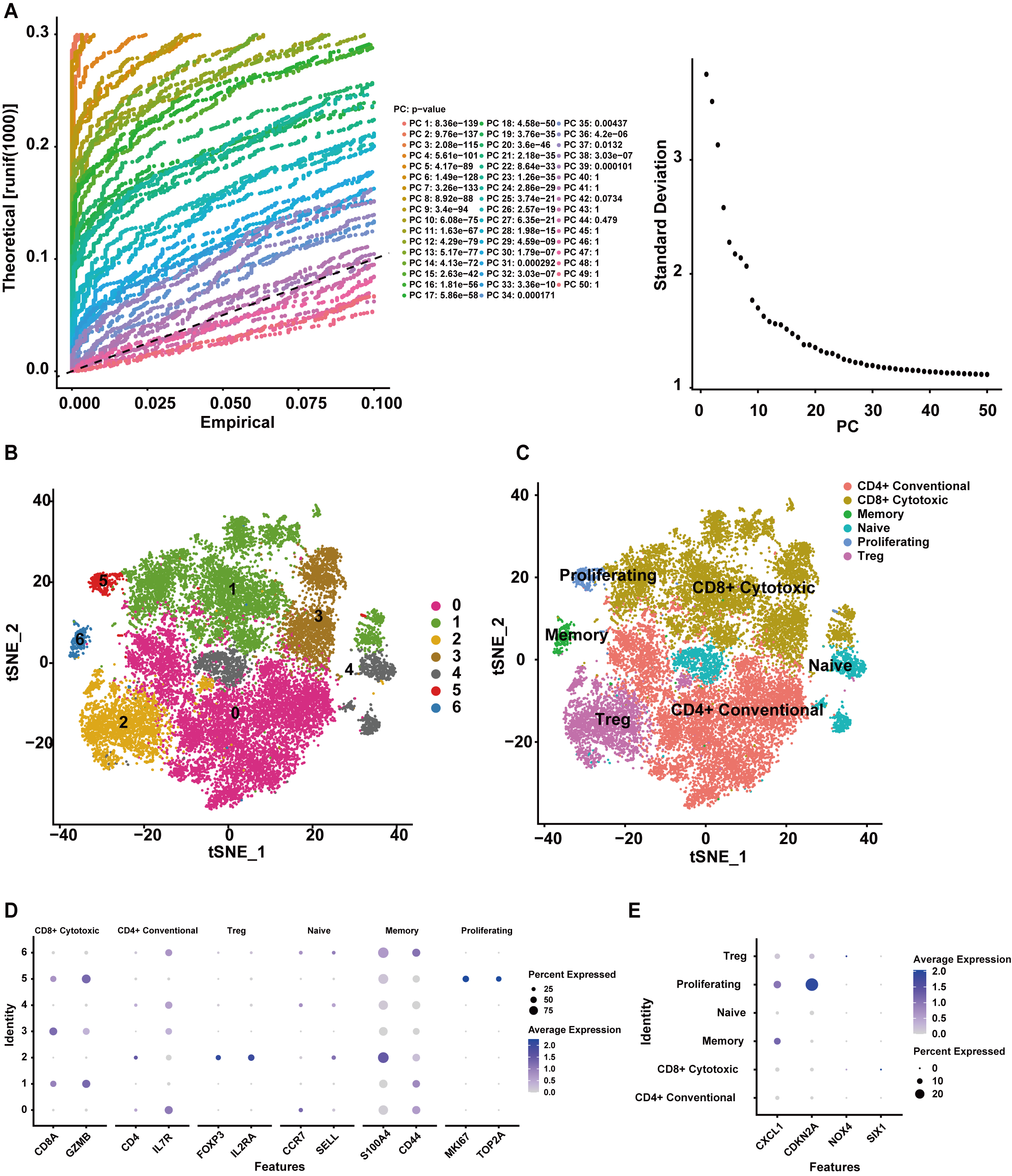

Supplement: Supplementary Figure 7 — Annotation of T-cell subpopulations and validation of expression levels. (A) PCA results of the single-cell dataset. Left panel: PCA result showing the dimensionality reduction process using principal component analysis; each line represents one principal component. Right panel: Scree plot, with the x−axis indicating principal components and the y-axis indicating standard deviation, which describes the dispersion or variability of the data. (B) Clustering analysis results. (C) Cell annotation results. (D) Expression of marker genes for T-cell subpopulation annotation. The x-axis shows marker genes, and the y-axis shows cell types. Larger and bluer circles indicate higher gene expression in the cells. (E) Expression of core genes in T−cell subpopulations. Dot plot showing expression levels of CXCL1, CDKN2A, NOX4, and SIX1 in T-cell subpopulations. Bluer color indicates higher expression level, and larger circle size indicates a higher proportion of cells expressing the gene. [file Image7.tif]

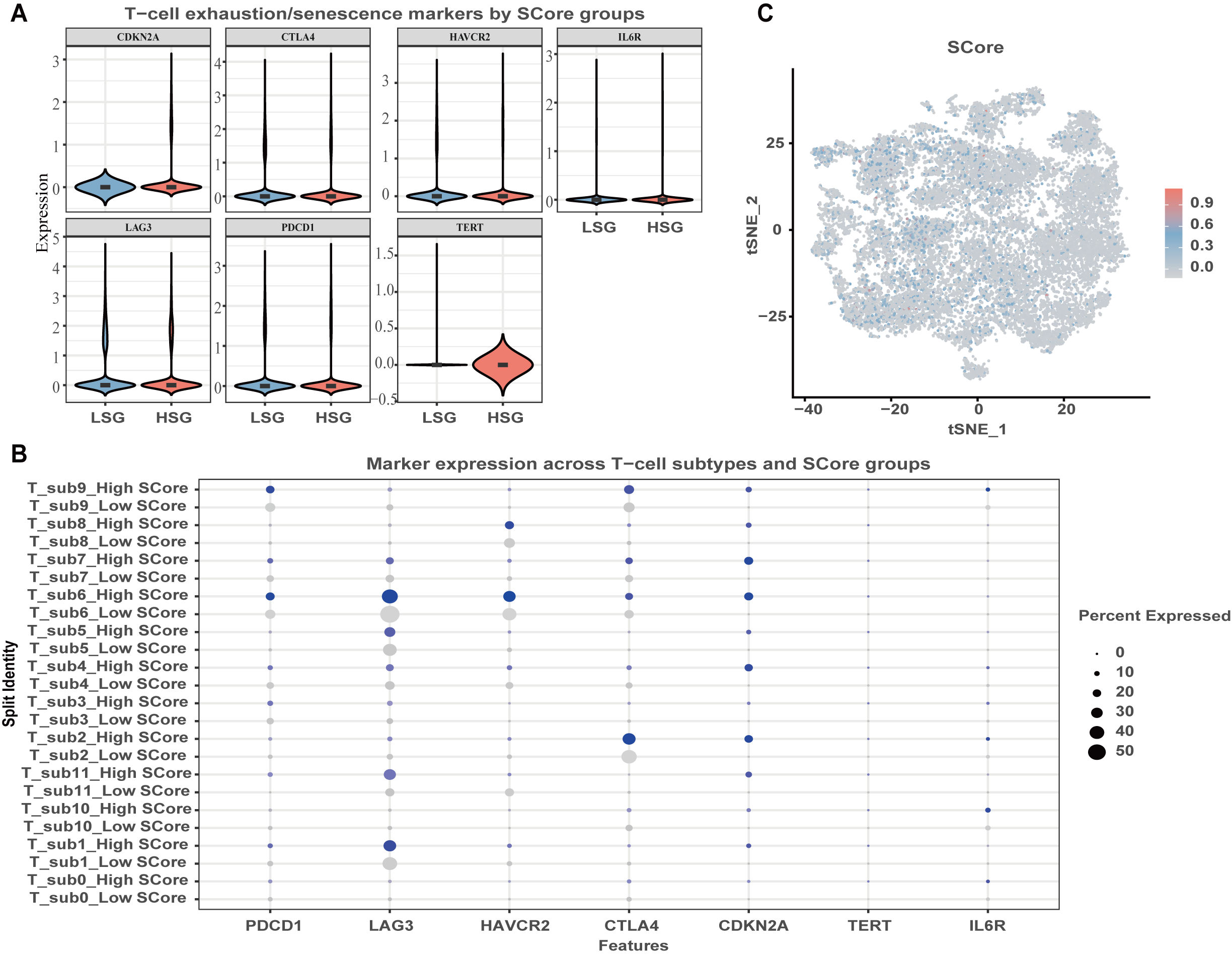

Supplement: Supplementary Figure 8 — SCore-associated T-cell expression states. (A) Differential expression of exhaustion/senescence markers in T cells between high- and low- SCore groups. Expression distributions in high-SCore versus low−SCore T cells. Blue represents the low−SCore group, and red represents the high−SCore group. (B) Dot plot of marker expression stratified by high/low SCore across different T-cell subpopulations. Color indicates average expression, and dot size represents the proportion of cells expressing the marker. (C) Distribution of T-cell SCore in the reduced dimensional space. Continuous distribution of T-cell SCore shown in t−SNE space, reflecting the spatial aggregation of cells with high functional scores within the population. [file Image8.tif]

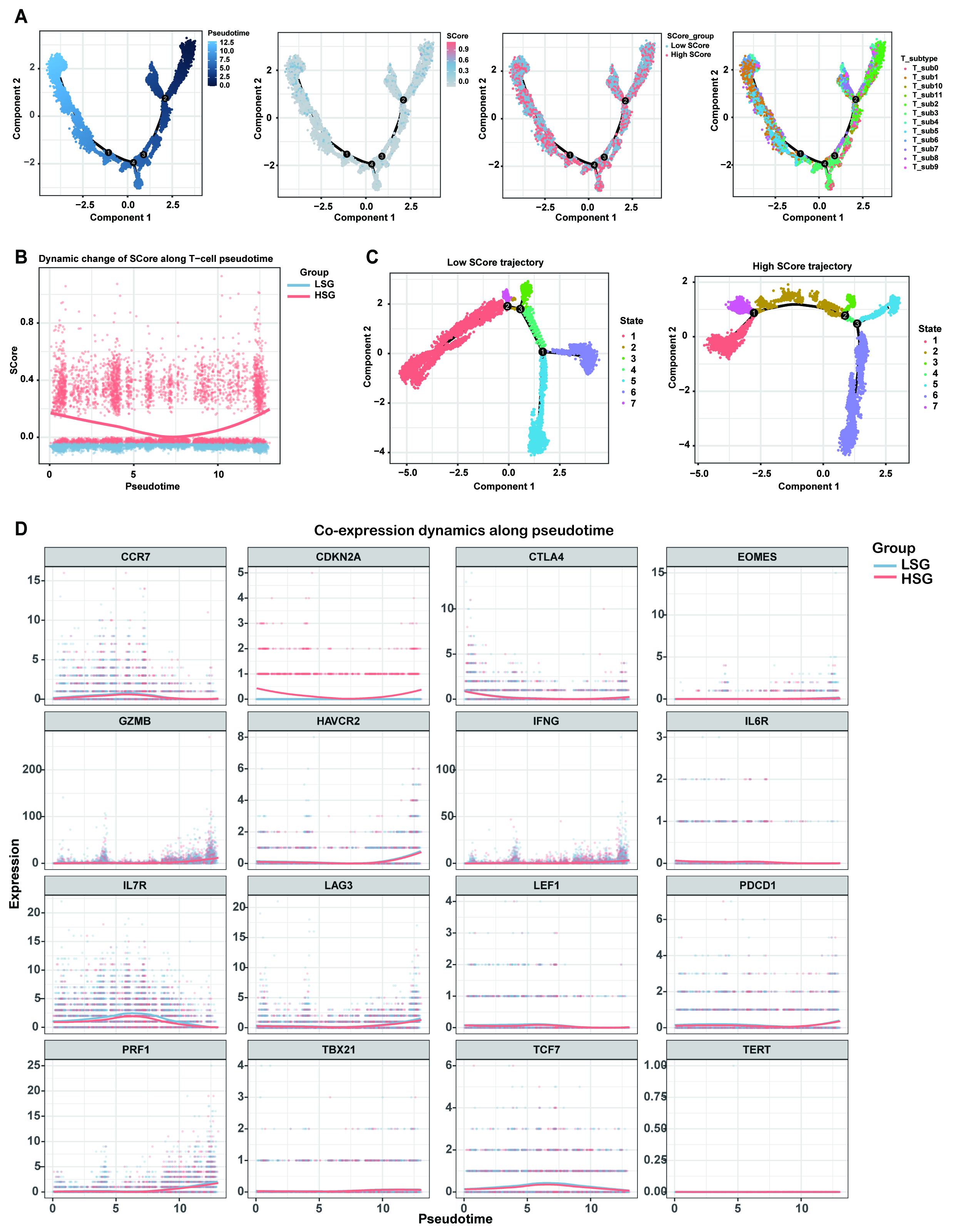

Supplement: Supplementary Figure 9 — SCore-associated pseudotime analysis of T cells. (A) Pseudotime trajectory of T cells and SCore mapping. Top left: Colored by pseudotime, showing the direction of differentiation progression. Top right: Colored by continuous SCore, showing the spatial distribution of SCore along the trajectory. Bottom left: Colored by SCore_group (High/Low), showing enrichment differences of high and low groups in different regions of the trajectory. Bottom right: Colored by T_subtype, displaying the distribution pattern of subpopulations along the differentiation path and potential branch preferences. (B) Dynamic changes along T- cell pseudotime. (C) Comparison of pseudotime trajectories between high- and low-SCore T−cell groups. (D) Co-expression dynamics of key markers along pseudotime. [file Image9.tif]

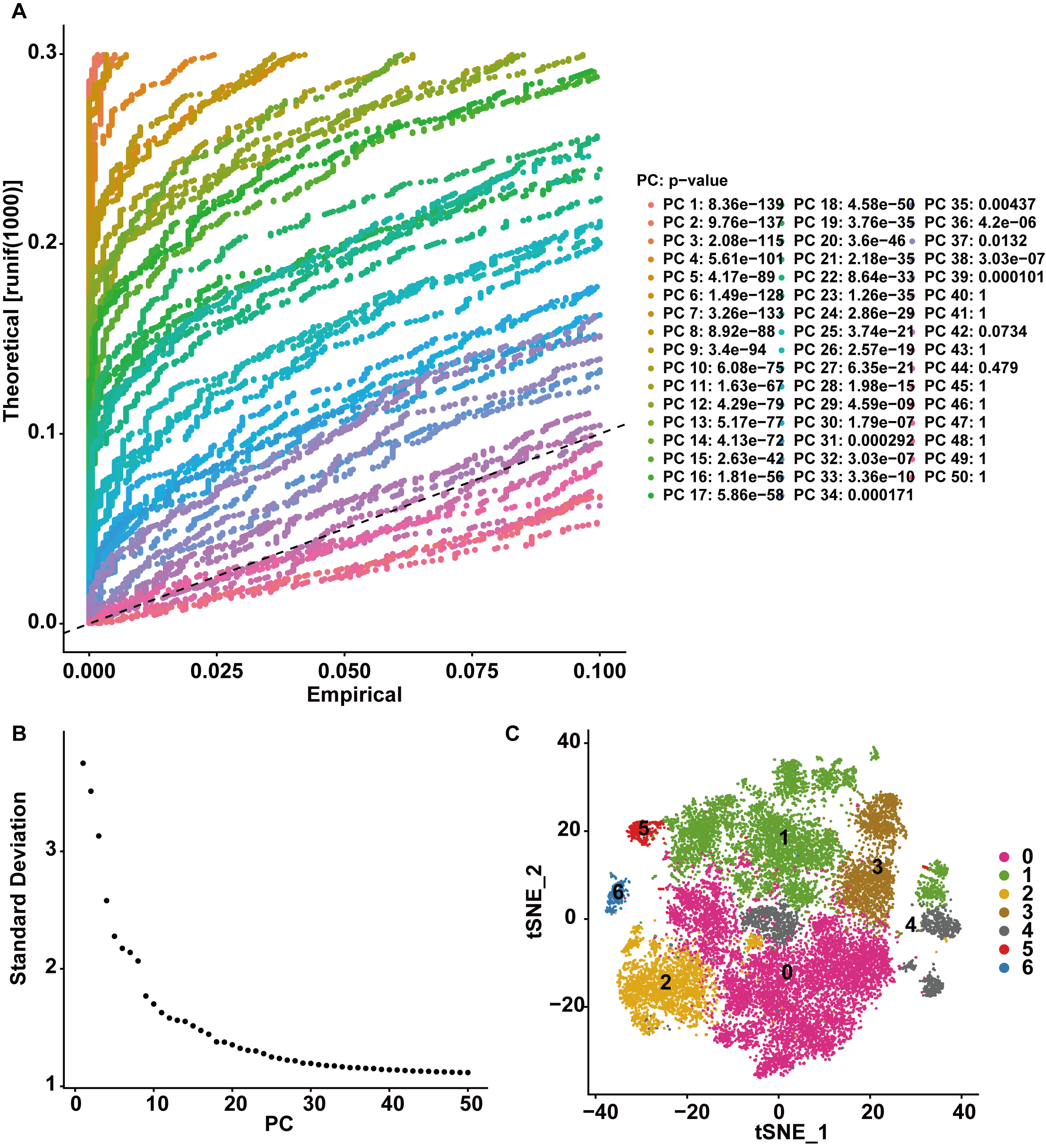

Supplement: Supplementary Figure 10 — Dimensionality reduction and subclustering analysis of T cells in the single-cell RNA sequencing dataset. (A) Principal component analysis (PCA) significance assessment of T cells using JackStraw analysis. (B) PCA scree plot showing the variance explained by each principal component in T cells. (C) t-distributed stochastic neighbor embedding (t-SNE) plot illustrating subclustering of T cells based on selected principal components. [file Image10.tif]

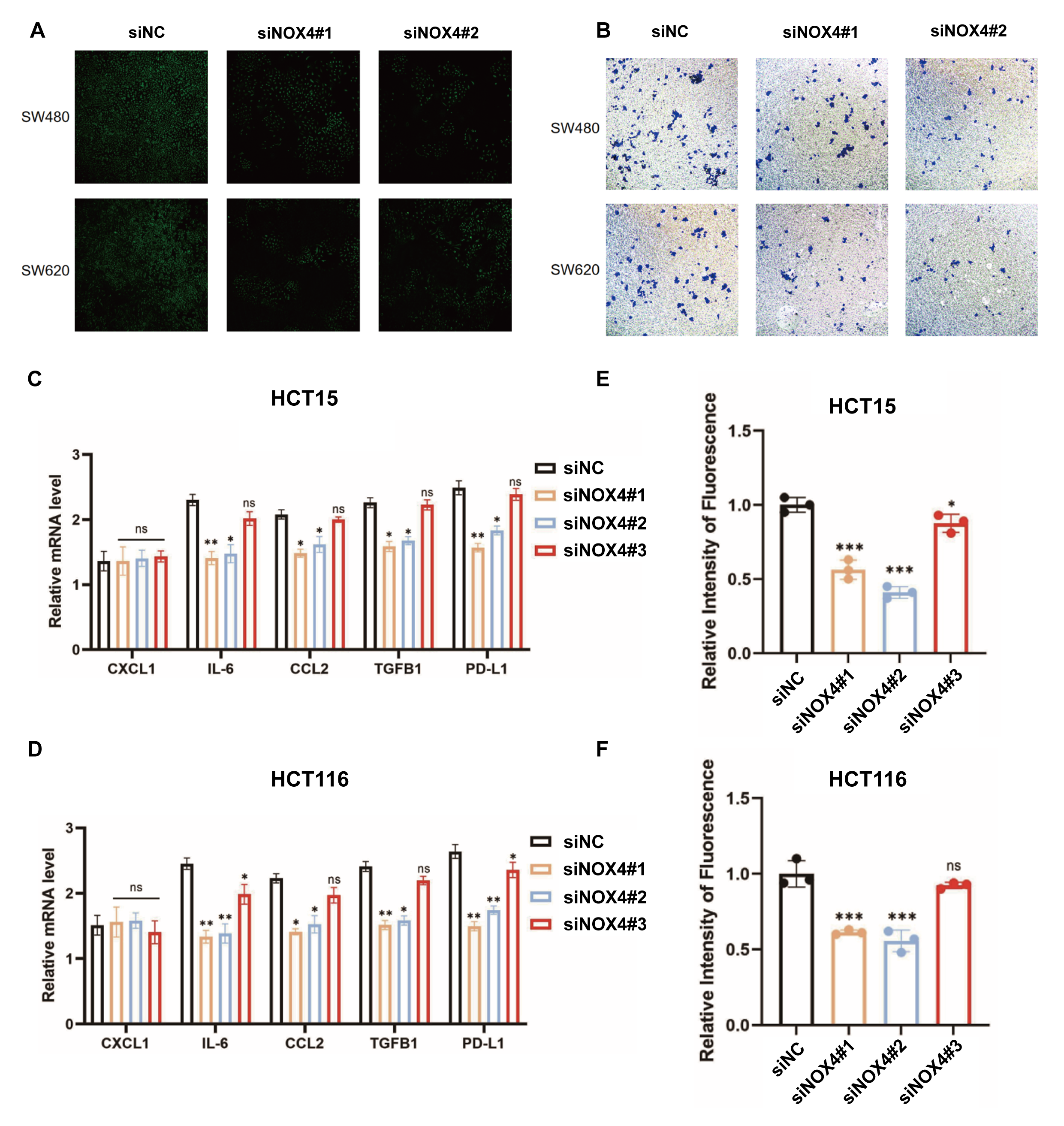

Supplement: Supplementary Figure 11 — Representative imaging and additional validation of NOX4 knockdown effects. (A) Representative fluorescence images showing intracellular ROS levels in SW480 and SW620 cells following NOX4 knockdown. (B) Representative images of Transwell migration assays in SW480 and SW620 cells after NOX4 knockdown. (C, D) qPCR analysis of IL-6, CCL2, TGF-β1, and PD-L1 mRNA levels following transient NOX4 knockdown in HCT15 and HCT116 cells. (E, F) Quantification of intracellular ROS levels in HCT15 and HCT116 cells after NOX4 knockdown. [file Image11.tif]
